# *Drosophila* Hox genes induce melanized pseudo-tumors when misexpressed in hemocytes

**DOI:** 10.1038/s41598-021-81472-5

**Published:** 2021-01-19

**Authors:** Titus Ponrathnam, Ravina Saini, Sofia Banu, Rakesh K. Mishra

**Affiliations:** 1 Centre for Cellular and Molecular Biology, Hyderabad, 500007 Telangana India; 2 Academy of Scientific and Innovative Research (AcSIR), New Delhi, India

**Keywords:** Cancer genetics, Haematological cancer

## Abstract

Hox genes are early determinants of cell identity along the anterior–posterior body axis across bilaterians. Several late non-homeotic functions of Hox genes have emerged in a variety of processes involved in organogenesis in several organisms, including mammals. Several studies have reported the misexpression of Hox genes in a variety of malignancies including acute myeloid leukemia. The Hox genes *Dfd, Ubx, abd-A* and *Abd-B* were overexpressed via the UAS-Gal4 system using *Cg-Gal4, Lsp2-Gal4, He-Gal4* and *HmlD3-Gal4* as specific drivers. Genetic interaction was tested by bringing overexpression lines in heterozygous mutant backgrounds of Polycomb and trithorax group factors. Larvae were visually scored for melanized bodies. Circulating hemocytes were quantified and tested for differentiation. Pupal lethality was assessed. Expression of *Dfd, Ubx* and *abd-A,* but not *Abd-B* in the hematopoietic compartment of *Drosophila* led to the appearance of circulating melanized bodies, an increase in cell number, cell-autonomous proliferation, and differentiation of hemocytes. Pupal lethality and melanized pseudo-tumors were suppressed in *Psc*^*1*^ and *esc*^*2*^ backgrounds while polycomb group member mutations *Pc*^*1*^ and *Su(z)12*^*3*^ and trithorax group member mutation *TrlR*^*85*^ enhanced the phenotype. *Dfd, Ubx* and *abd-A* are leukemogenic. Mutations in Polycomb and trithorax group members modulate the leukemogenic phenotype. Our RNAseq of *Cg-Gal4* > *UAS-abd-A* hemocytes may contain genes important to Hox gene induced leukemias.

## Introduction

Life comes in a variety of body forms. Despite this variety, there is similarity at the genetic and molecular level in the developmental mechanisms that lead to this variety across species. For example, despite the evolutionary distance between vertebrates and *Drosophila*, many organ and tissue types show a degree of homology with each other and many key developmental pathways governing their development and function are conserved. The hematopoietic system is no exception. Hemocytes of *Drosophila* resemble the myeloid lineage of blood cells^1^. The most abundant cells, plasmatocytes, are the equivalent of macrophages and are involved in a variety of processes such as responses to pathogens, removal of apoptotic cells, deposition of the extracellular matrix during embryonic development, etc.^2^ The next most abundant cells are Crystal cells, specialized to induce myelinization reactions in the presence of pathogens and wound healing^3^, resemble the granulocytes, and contribute about four per cent of the blood cells. Lamellocytes are the least abundant population of blood cells, usually only appearing in circulation upon the larva being challenged by any object too large to be cleared off by the macrophages, such as the eggs of a parasitoid wasp^4,5^. Hematopoiesis in *Drosophila* occurs in during the larval stage in the Lymph Gland (LG)^6^.

The conservation between vertebrate and *Drosophila* hematopoiesis extends to their genetic basis^7^. For example, *serpent* is orthologous to GATA 1–3^8^. *Drosophila u-shaped* is orthologous to members of the Friend of GATA (FOG) family^9^. Signaling pathways involved in regulating hematopoiesis are similarly conserved. Jagged-1, the vertebrate homologue of Serrate and ligand of Notch, is presented by stromal cells to regulate Hematopoietic stem cells to regulate their proliferation and survival^10^, similar to the role performed by Serrate presented by the Posterior Signaling Centre (PSC)^11^, a set of regulatory cells at the posterior end of the LG, via cytonemes^12,13^. Mutations in JAK2 can lead to leukemogenesis in vertebrates^14^, similar to mutations in *Hopscotch*^15,16^*.* The Toll pathway is also conserved, playing a major role in innate immunity in both vertebrates and flies^17^.

Leukemias have been modeled extensively in flies^18^, including the leukemogenicity of human fusion proteins^19,20^. Melanized masses, referred to as pseudotumors, were identified as encapsulations caused by differentiated lamellocytes^21,22^. Melanogenesis is a central to insect innate immunity which leads to the formation of cytotoxic reactive oxygen and nitrogen species, for clearing pathogens as well as parasites^23^. Mutations in genes such as *Toll* and *Hop* lead to aberrant lamellocyte differentiation, hemocyte over proliferation, as well as melanotic tumors^16,24^. Melanized masses may be hematogenic in origin resulting in circulating masses, or as a result of mutations that lead to tissues being melanized in an “autoimmune” manner^25–27^.

One aspect of vertebrate hematopoiesis that has not been demonstrated in *Drosophila* is the role of Hox genes. Hox genes are well known for their conserved role in body axis formation across all bilaterians^28^, but also play roles in vertebrate hematopoiesis^29^, autophagy^30^, as well as cell proliferation, differentiation, migration and apoptosis^31^. Hox genes are transcribed in HSCs as well as lineage progenitors and are suppressed in differentiated blood cells^32–36^. Overexpression models show blockages in certain stages of development, expansion of HSCs, the circulation of blast cells, etc.^37–42^ For example, *Hoxa7* and *Hoxa9* have been shown to have a role in the development of hematopoietic progenitors of different lineages in mice. In *Drosophila, Antennapedia* is required for maintenance of collier expression and marks the PSC^43^. The expression domain of Ubx forms the posterior extent of the lymph gland, with the dorsal vessel developing into an LG like tissue in *Ubx*^−^ larvae^44,45^.

The expression of genes of the *Hox* cluster during, and after development is regulated by two chromatin remodelers, Polycomb and trithorax group (PcG and trxG) of proteins, which were discovered as transcriptional repressors (PcG) and activators (trxG) of *Hox* genes in *Drosophila*^46^. Later, these proteins were shown to regulate many biological processes such as cell fate and lineage, cellular memory, stem cell function, and tissue homeostasis in cell lines and mouse models^47–50^. The deregulation of Hox genes via Polycomb or trithorax proteins can lead to leukemogenesis by mis-regulation of hematopoiesis. Furthermore, PcG members EZH2, a human homolog of *Drosophila* E(z) protein, EED (Esc in *Drosophila*), SUZ(12) (*Drosophila* Su(z)12) and BMI-1(homolog of *Drosophila Psc*) have been shown to have a role in different cancers in knock out studies carried out in cell lines as well as in mouse model^51–54^. Mixed Lineage Leukemia (MLL), the human homolog of *Drosophila* Trithorax (Trx) protein, regulates *Hoxa* expression in HSCs. MLL is a frequent fusion protein partner in acute leukemia^55^. Evidence for the role of PcG and trxG genes in regulating HSC development in *Drosophila* remains largely to be explored^56,57^.

Melanized pseudo tumors are a hallmark of aberrant hematopoiesis in *Drosophila.* In this study, we show that overexpression of the Hox genes, *Dfd, Ubx* and *abd-A* in blood cells not only leads to melanized pseudo-tumors, but also to a significant increase in blood cell number and the induction of lamellocyte differentiation. Further, we present genetic evidence to show the role of PcG members, *Psc* and *Esc,* in the melanized pseudo-tumor formation induced by Hox genes. These findings will be helpful in understating the biological events associated with leukemia in humans, which may open new possibilities of markers and therapy. Some potential events may be represented in our RNAseq data obtained from hemocytes of the *Cg-Gal4* > *UAS abd-A* genotype.

## Results

### Tumor phenotype correlates with the tissue specificity and strength of the driver

**Figure 1:**
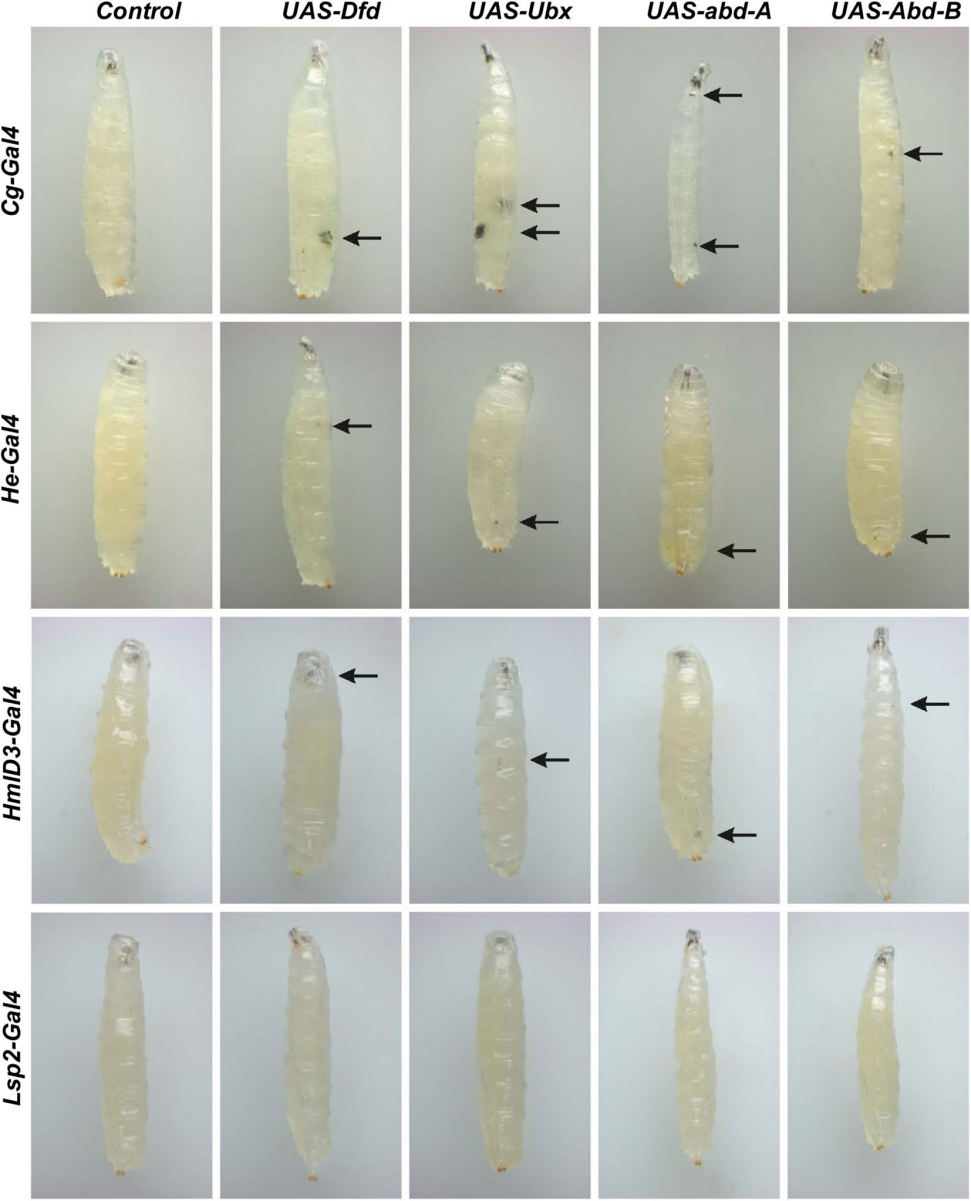
Larvae with subcutaneous tumors. *Dfd, Ubx, abd-A* and *Abd-B*, when expressed under the drivers *Cg, He* and *HmlD3* lead to melanized bodies in the viscera.

**Figure 2:**
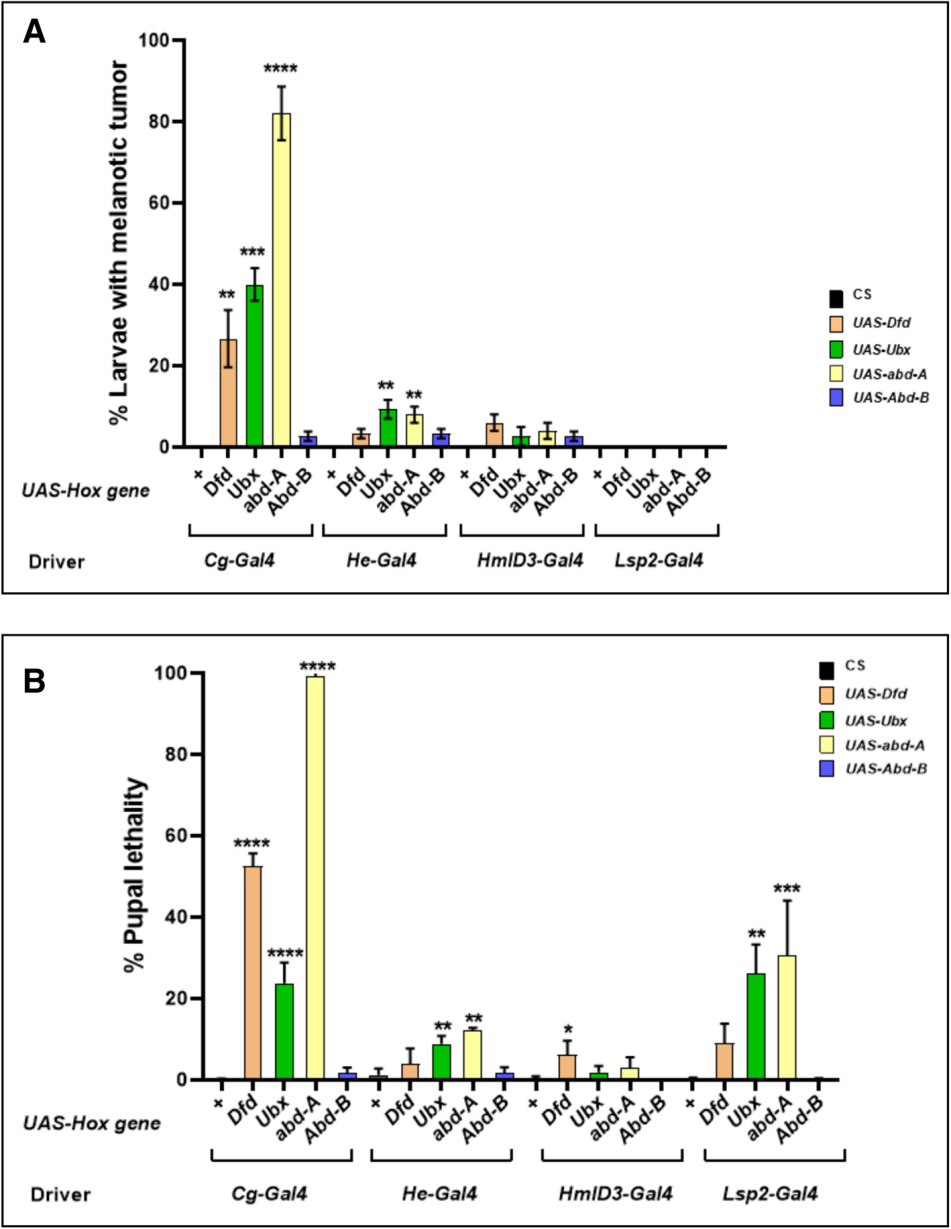
Tumor phenotype in larvae and pupal lethality. (**A**) The size and penetrance of melanized bodies was maximum when expressed under *Cg*, while tumors do manifest when *He* and *HmlD3* are used, they are much rarer and smaller. Expression under *Lsp2*-*Gal4* does not lead to the formation of such bodies. (**B**) Percentage of pupal lethality, indicated by larvae that fail to eclose. When Cg-Gal4 drives the genes *Dfd, Ubx, abd-A* and *Abd-B* do cause lethality, so does expressing them in the fatbody under *Lsp2*-*Gal4*. Driving these genes in the blood cells (*He*-*Gal4* and *HmlD*3-*Gal4*) leads to a much lower penetrance of this phenotype.

In *Drosophila*, the *collagen-Gal4* (*Cg-Gal4)* driver induces the strong expression UAS tagged genes in the fatbody as well as in the hematopoietic system^58^. The different UAS Hox genes lines, *Dfd, Ubx, abd-A* and *Abd*-*B,* when brought under the *Cg-Gal4* driver, induced melanized pseudo-tumors in larvae. This phenotype manifested in 26% of *Cg-Gal4* > *UAS Dfd* larvae, 60% of *Cg* > *Ubx* larvae, 82% of *Cg-Gal4* > *UAS abd-A* larvae and 4% of *Cg-Gal4* > *UAS-Abd-B* larvae (Figs. 1, 2A, B and Supplementary Table S3).

We then used the *Hemese-Gal4 (He-Gal4)* driver, which exhibits negligible expression in the LG and drives expression in about 80% of circulating hemocytes^59^, and the *HemolectinD*3*-Gal4 (HmlD*3*-Gal4)* driver, which expresses in the cortical region of the lymph gland as well as in mature circulating hemocytes. While melanized pseudo tumors were observed in these genotypes, they appeared smaller and the penetrance of the phenotype was very low, manifesting in 3% of *He-Gal4* > *UAS Dfd,* 6% in *HmlD*3*-Gal4* > *UAS Dfd,* 9% in *He-Gal4* > *UAS Ubx,* 2% in *HmlD*3*-Gal4* > *UAS Ubx,* 8% in *He-Gal4* > *UAS abd-A*, 4% in *HmlD*3*-Gal4* > *UAS abd-A,* 3% in *He-Gal4* > *UAS Abd-B* and 2% in *HmlD*3*-Gal4* > *UAS Abd-B* (Figs. 1, 2A, B and Supplementary Table S3)*.* Over-expression of *Hox* genes with the *He-Gal4* driver always showed a higher penetrance of the phenotype when compared to *HmlD*3*-Gal4*. Lamellocytes are responsible for the encapsulation mechanism in combating an immune challenge, and they do not express *Hemolectin*. The low penetrance of the phenotype in *HmlD*3*-Gal4* could be due to a lack of expression in lamellocytes^52,60^. Also, *Hemolectin* does not express in the medullary zone of the lymph gland, where cell proliferation and differentiation take place^61^. It shows the phenotype is associated with active proliferation and differentiation of hemocytes of developing larvae. To test that the phenotype was not due to expression of the Hox genes in the fatbody (as *Cg-Gal4* expresses in both blood cells as well as the fatbody) we over-expressed these genes using the fatbody specific driver *Lsp2-Gal4. Lsp2-Gal4* functions in L3 larval fat bodies^62^. No melanized spots were observed in such larvae, indicating that the pseudo-tumor phenotype is not induced by the misexpression of Hox gene in the fatbody.

### Tumor phenotype is co-related with lethality at the pupal stage

We also noticed a significant level of pupal lethality when *Hox* genes were mis-expressed in these conditions. Pupal lethality with the *Cg-Gal4* driver was highest when it drives *UAS-abd-A* (99%). *Cg-Gal4* > *UAS Dfd* (53%) and *Cg* > *Ubx* (24%) also show an increased lethality at pupal stage. It was negligible in *Cg-Gal4* > *UAS-Abd-B* (2%). We observed lethality when the same genes were over expressed in the fatbody with *Lsp2-Gal4.* However, *Lsp2-Gal4* driven Hox expression induced lethality was lower compared to *Cg-Gal4* driven Hox expression induced lethality, except in the case of *Lsp2-Gal4* > *UAS-Ubx*. But it must be noted that it was greater than that induced by the blood specific drivers used by us. Pupal lethality with *Lsp2-Gal4* driver was observed 9% in *Lsp2-Gal4* > *UAS Dfd,* 26% in *Lsp2-Gal4* > *UAS Ubx* and 31% in Lsp2*-Gal4* > *UAS abd-A*. It has previously been shown that aberrant blood cells can induce pupal lethality^63^. However, while we did observe some pupal lethality when the Hox genes were expressed under *He* and *Hml*, the lethality was most prominent in when the *Cg-Gal4* or *Lsp2*-Gal4 drivers were used (Fig. 2B, Supplementary Table S4) which supports the earlier report suggesting that Hox genes are repressors of autophagy in the fatbody^30^. Thus, while we do observe lower levels of lethality with blood specific drivers, since the expression of Hox genes in the fatbody does indeed induce lethality, the greater lethality when *Cg-Gal4* is used may be due to the concomitant expression induced in the fatbody as well as blood cells.

### Hox genes over-expression induces hemocyte proliferation and differentiation

**Figure 3:**
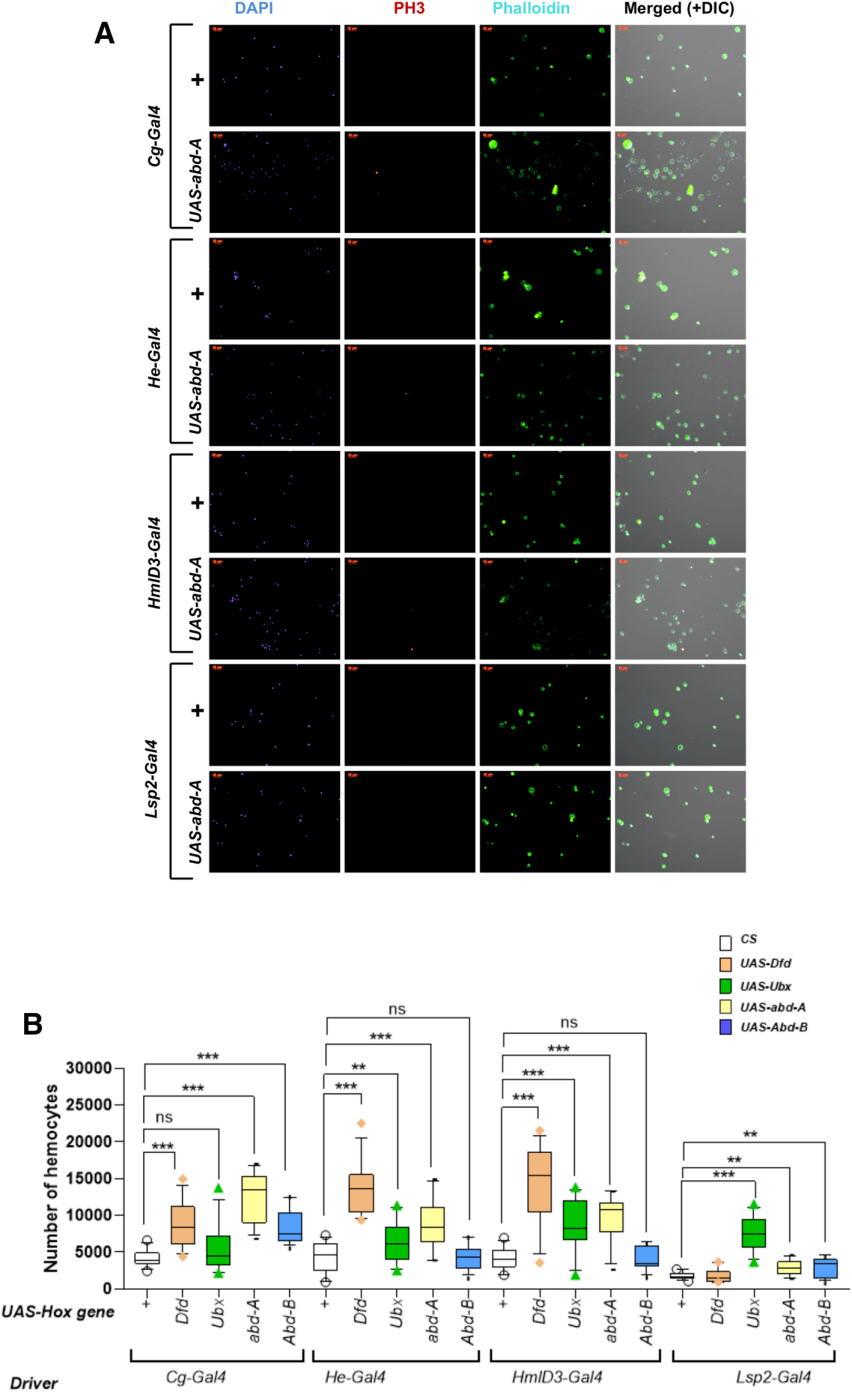

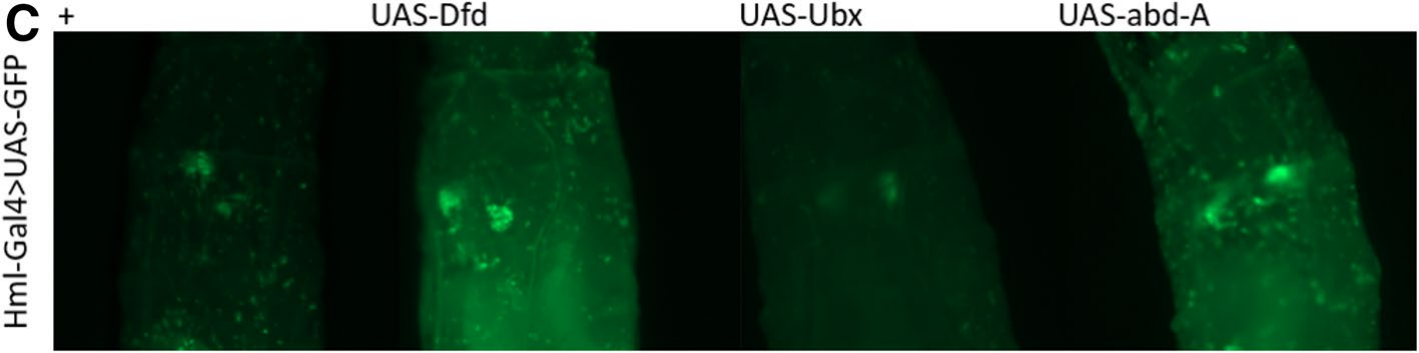
Cell proliferation and quantification of hemocytes. (**A**) Anti-PH3 staining for comparative study of cell proliferation with over-expression of *abd-A* gene driven by *Cg-Gal4, He-Gal4, HmlD*3*-Gal4 and Lsp2-Gal4*. Phalloidin iFluor-488 was used to stain actin filaments. PhosphoHistone3+ nuclei appear when these genes are expressed in the blood cells*, indicatin*g that some of the increase in cell number maybe due to cell autonomous proliferation. (**B**) The number of circulating hemocytes increases significantly when *Dfd* is over expressed using *Cg-Gal4* (*p* = 0.0003), *He-Gal4* (*p* < 0.0001) and *HmlD*3*-Gal4*(*p* < 0.0001), *Ubx* when over expressed with *Lsp2-Gal4* (*p* < 0.0001) and *HmlD*3*-Gal4* (*p* = 0.0011), *abd-A* when over expressed with *Cg-Gal4* (*p* < 0.0001), *Lsp2-Gal4* (*p* = 0.0028), *He-Gal4* (*p* = 0.0018) and *HmlD*3*-Gal4* (*p* = 0.0002), *Abd-B* when over expressed with *Cg-Gal4* (*p* < 0.0001). (**C**) *UAS-Dfd, USA-Ubx* and *UAS-abd-A* were over expressed in a *HmlD3-Gal4, UAS-GFP* background, to test whether overexpression of the genes led to the premature release of cells resident in the cortical zone of the lymph gland. The cortical zones of the over expression larvae appeared to be GFP positive.

Change in the number of cells and types of cells become important considering the phenotype observed upon misexpression of Hox genes. We quantified the number of blood cells in our overexpression lines using a modified version of established methods^64,65^. When expressed by blood specific driver*, Dfd, Ubx* and *abd-A* led to a significant increase in the number of circulating hemocytes (Fig. 3A, B, Supplementary Tables S5–S8) and many proliferating cells show co-localization of hox over-expression and PH3 (Supplementary Fig. 2A–D). Interestingly, while the penetrance of melanized spots was lower, blood specific drivers showed a larger number of blood cells (Fig. 3B). Under the control of, *Lsp2,* the fatbody exclusive driver, however, *Ubx* and *abd-A* gave a significant increase in hemocyte number*,* despite them not manifesting melanized spots. Our results show that melanized spots (or pseudo-tumors), which have been reported as the hallmarks of a “leukemia-like” phenotype in *Drosophila,* may not reflect an actual increase in hemocytes. Additionally, studies have used the strong driver *Cg-Gal4,* which drives expression in fatbody as well as the blood cells^25,66,67^. As our results show that the ectopic expression of genes in the fatbody may indeed lead to an increase in circulating hemocytes (Fig. 2B), future studies should take care while interpreting results obtained with *Cg-Gal4*. It may also be that the number of circulating cells when we expressed the hox genes under *Cg-Gal4* may be due to circulating cells being trapped in the melanized pseudo tumors.

It has previously been shown that cells of the LG do not enter circulation until the onset of metamorphosis^68^. However, *Hml* and *Cg* express in the cortical region of the LG, while *He*-*Gal4* expresses negligibly in the LG. Thus, the question arose as to whether the increase in cell number was due to an increase in cell proliferation at the LG or were circulating cells proliferating in a cell-autonomous manner. Hence, we checked for the presence of the mitotic cell marker PH3. We observed cells positive for PH3, when Hox genes were expressed in the blood cells, and not when expressed exclusively in the fatbody (Fig. 3A, Supplementary Fig. 1A–D). Unlike previous reports, we did not find proliferative cells in our control experiments^69^. This may be due to a loss of cells in our preparations or more robust immunostaining on our part. Thus, while we cannot rule out the possibility that LG cells contribute to this increase, at least a fraction of the increase takes place due to the cell autonomous division of Hox overexpressing cells. As cells of the LG could potentially be released prematurely into circulation on account of Hox gene over expression, we checked for the integrity of the LG by overexpressing *UAS-Dfd, UAS Ubx* and *UAS-abd-A* in an *Hml-Gal4, UAS-GFP* background. LGs remained intact 96hrs post egg lay (Fig. 3C).

**Figure 4:**
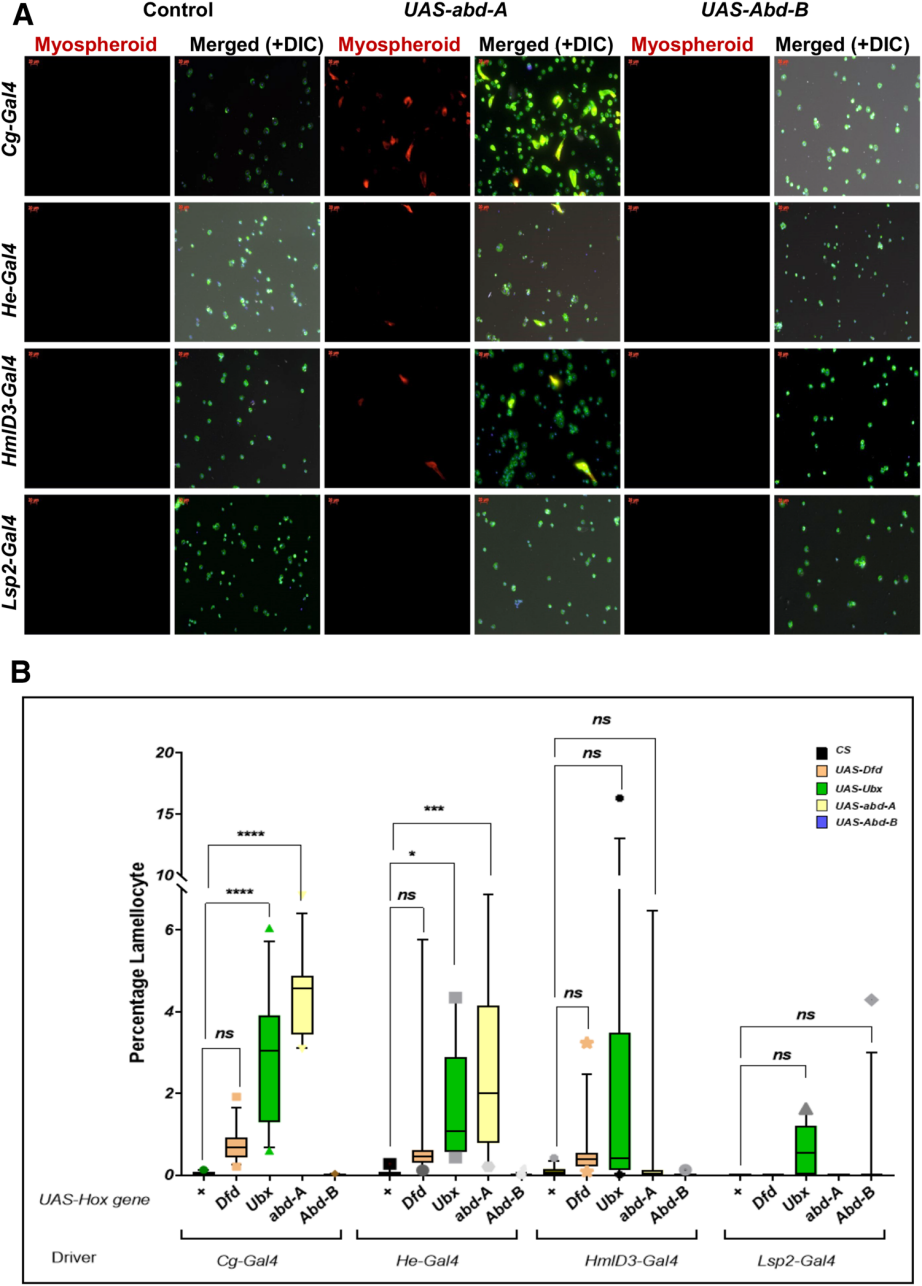
*Myospheroid* staining and quantification of lamellocytes. (**A**) When *Dfd, Ubx, abd-A,* but not *Abd-B* are driven in blood cells (under *Cg-Gal4*, *He*-*Gal4* or *HmlD*3-*Gal4*), but not in the fatbody (*Lsp2*-*Gal4*), large, dorsoventrally flattened cells begin to appear in circulation. These stain positive for mys*.* Some circulating plasmatocytes also appear to mys+. This indicates that they might be in the process of differentiating. (**B**) Percentage of lamellocyte is plotted on Y-axis. Ubx over-expression with *Cg-Gal4* (*p* < 0.0001) and *He-Gal4* (*p* = 0.0455) has a significant increase while *Lsp2-Gal4* and *HmlD*3*-Gal4* do not show any significant increase. abd-A with *Cg-Gal4* and *He-Gal4* has comparatively high percent of increase to control as well as Ubx over-expressed.

While imaging the blood cells, we noticed that there were larger, flattened cells in circulation, reminiscent of lamellocytes. To test whether they were *bona fide* lamellocytes, we stained the hemocytes for the lamellocyte marker myospheroid^70^ (Fig. 4A, Supplementary Fig. 1A–D). Control larvae infrequently showed the presence of lamellocytes. In our overexpression lines, however, we noticed that several cells appeared morphologically as lamellocytes and were mys+. Some plasmatocytes also stained positive for mys. None of the plasmatocytes in the control flies or those overexpressing Abd-B were positive for mys. Previous reports have suggested that circulating plasmatocytes may differentiate into lamellocytes^71,72^. Thus, it may be that these circulating mys+ plasmatocyte like cells are differentiating into lamellocytes. However, *Lsp2-Gal4* > *UAS Ubx* also had a significant number of lamellocytes. This is in keeping with reports that signals from the fat body can drive lamellocyte differentiation^73,74^. Thus, we speculate that these cells, upon Hox overexpression, are pushed toward the lamellocyte fate (Fig. 4A,B).

### Effect of PcG and trxG genes

**Figure 5:**
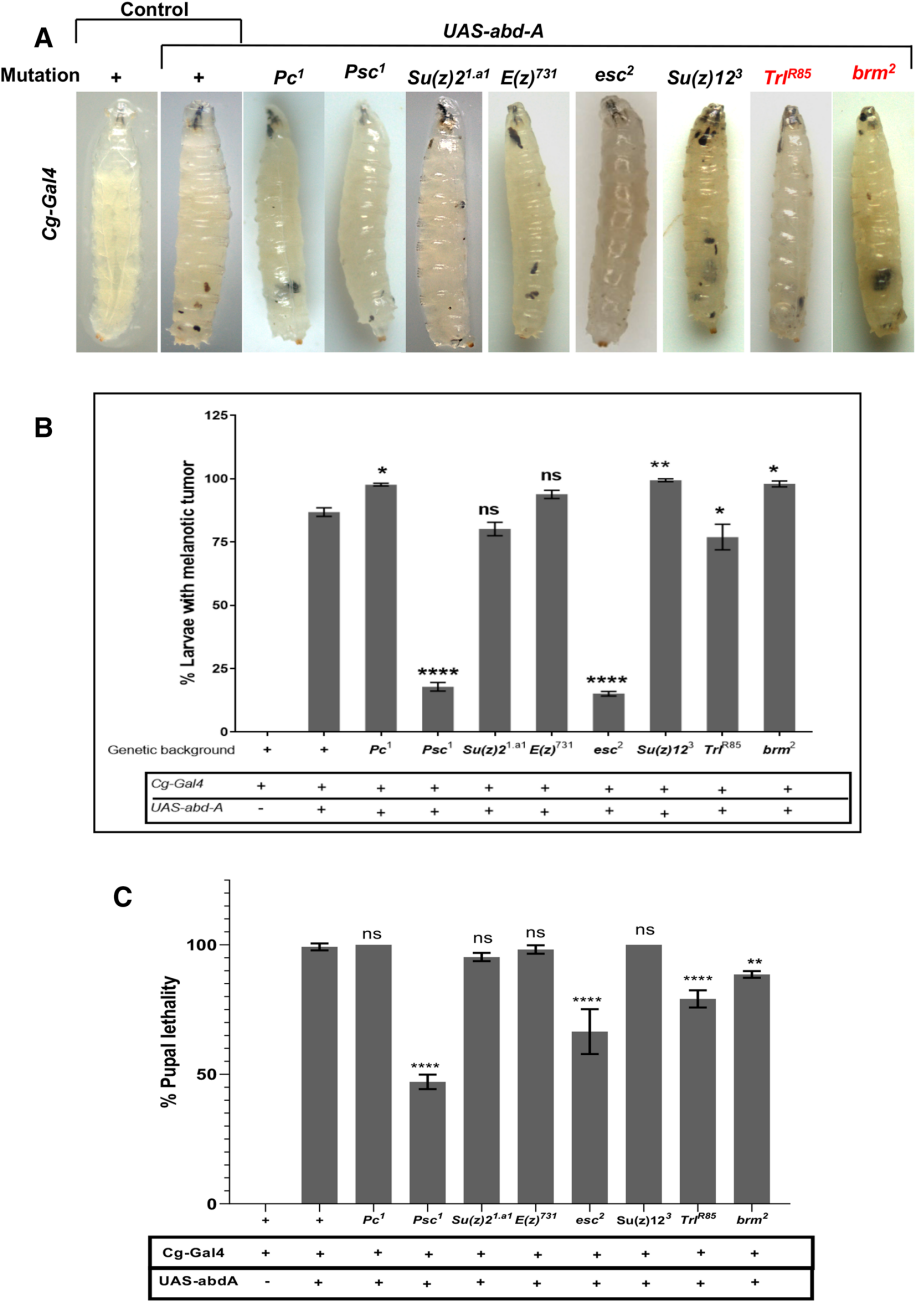

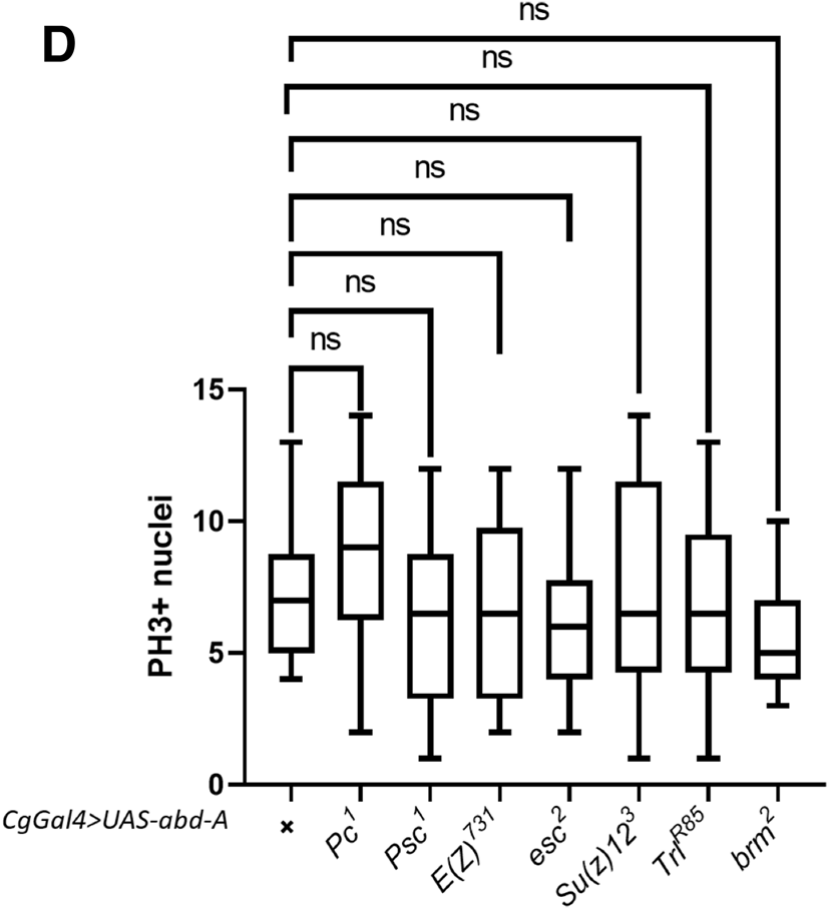
Effect of PcG and trxG mutations on the abd-A induced tumor phenotype. (**A**) Effect of PcG and trxG on subcutaneous melanotic tumor formation. (trxG members have been highlighted in red in 5A) Over-expression of abd-A gene is driven by Cg-Gal4. Most of PcG and trxG mutants have effect on the phenotype. Polycomb mutants, esc^2^ (PRC2 member) and *Psc*^*1*^ (PRC1 member) show a decrease in severity of phenotype (size and numbers of melanotic body). *Su(z)12*^*3*^ and *Pc*^*1*^ have increased severity. *Brm*^*2*^*, Trl*^*R85*^*, Su(z)2*^*1.a1*^ and *E(z)*^*731*^ do not show any change in severity compare to Cg-Gal4 driven abd-A over-expressed individual. (**B**) Comparative quantification of melanotic tumor formation phenotype in abd-A over-expressed (driven by Cg-Gal4) individual in different PcG and trxG background. Percentage of tumor showing individuals is plotted on Y-axis (no. of animal screened is > 80 in each case, error bars represent the standard error). Phenotype is rescued in *esc*^*2*^ (*p* < 0.0001)*, Psc*^*1*^(*p* < 0.0001), and *Trl*^*R85*^ (*p* = 0.0223) mutants while *Pc*^*1*^ (*p* = 0.0109),*Su(z)12*^*3*^ (*p* = 0.0028) and *Brm*^*2*^ (*p* = 0.0088) have a significant increase in melanotic tumor formation. (**C**) Percent pupal lethality in PcG and trxG mutant background. Psc^1^ (*p* < 0.0001), esc^2^ (*p* < 0.0001), brm^2^ (*p* = 0.0042) and *Trl*^*R85*^ (*p* < 0.0001) show decrease in pupal lethality. (**D**) Number of PH3 positive larvae in mutant backgrounds, Compared to *Cg-Gal4* overexpression of abd-A alone, the overexpression did not significantly alter the number of PH3+ hemocytes in *Pc*^*1*^ (*p* = 0.2554)*, Psc*^*1*^ (*p* = 0.1275)*, E(Z)*^*731*^ (*p* = 0.7907)*, esc*^*2*^ (*p* = 0.3282), *Su(z)12*^*3*^ (*p* = 0.8642)*,* and *Trl*^*R85*^ (*p* = 0.8975) backgrounds.

PcG members are known to function primarily through two distinct complexes, PRC1 (consisting of *Pc, Psc, Su(z)2* and *Sce*) and PRC2 (consisting of *E(z), Su(z)12, Esc* and *Caf 1-55*)^75^. Members of the PcG and trxG have been shown to have a role in hematological malignancies in different clinicopathological data in leukemic patients and mice models^76,77^. To determine their role in melanized pseudo-tumor formation in flies, we over-expressed abd-A using *Cg-Gal4* in the background of different PcG and trxG mutants. We selected *Psc*^*1*^, *Pc*^*1*^*, Su(z)2, Su(z)12, E(z)* and *esc2* from the PcG and *brm*^*2*^*, Trl* from the trxG. Melanotic pseudo-tumor phenotype was used in our study to assay the effect of the mutants as it is convenient and robust. All experiments were performed in biological triplicates. The PcG mutants *Pc*^*1*^*, Su(z)12*^*3*^*,* and trxG member *brm*^*2*^ showed an increase in melanotic body formation (Fig. 5A,B and Supplementary Table S9), and enhanced the phenotype up to 100%. *Pc*^*1*^ and *Su(z)12*^*3*^ not only enhanced the penetrance (percentage phenotype showing larvae) but showed an increase in severity (scored as number and size of the black spots) compared to abd-A over-expressed in absence of mutants (Fig. 5A). Pc is a core protein of PRC1 and plays a role in negative regulation of its target genes. Su(z)12, a member of the PRC2 similarly enhances the pseudo tumor phenotype. Our results indicate these proteins might suppress genes involved in the immune cascade. Surprisingly, E(z) does not show any significant effect on penetrance. Su(z)2 also does not significantly affect the pseudo tumor phenotype or pupal lethality. On the other hand, *esc*^*2*^ (PRC2 member) and *Psc*^*1*^ (PRC1 member) showed a significant decrease in penetrance 15% and 17% respectively (Fig. 5B, Supplementary Table S9). The severity of the phenotype is also significantly reduced in both the mutant background (Supplementary Fig. 3A,B). These results indicate that genes involved in melanotic pseudo-tumor causing phenotype might be the target of the Esc and Psc proteins. Although it has been shown that Esc-E(z) complex is a thousand times effective compared to *E(z)* alone^78,79^, our results suggest that Esc regulates its targets independent of *E(z)* activity or, for that matter, any other member of the PRC2 complex in the observed phenotype. Similarly, Psc mutation rescued the phenotype. We tested whether bringing our overexpression in the PcG and trxG backgrounds affected the number of PH3 positive nuclei. We did not observe a significant change (Fig. 5D, Supplementary Table S12). As the average number of PH3+ nuclei in *cg-Gal4* > *UAS abd-A* larvae was 0.08% of the average of total hemocytes, it may be that the total number of dividing nuclei are too few to significantly differ.

### Effect of PcG mutants on the melanized pseudo-tumor related pupal lethality

To test the effect of mutations on pupal lethality, L3F larvae from each combination, which manifested melanized pseudo-tumors, were transferred to fresh vials and allowed to pupate and eclose. Larvae from overexpressed *abd-A* (driven by *Cg-Gal4*) with melanotic body showed up to 99% lethality at the pupal stage. Further, we checked pupal lethality in mutant background. Since all mutants are maintained over balancers (Table S2), we selected overexpressed progenies without balancer to confirm mutant in the same progeny and transferred them in new food vials. Pupal lethality in *Su(z)12*^*3*^*, Pc*^*1*^ and *Su(z)2*^*1.a1*^ was always 100% while we could get a few survivors from *Cg-Gal4* > *UAS abd-A* (Figs. 3C, 5C, Table S10). A decline in lethality was seen in *Psc*^*1*^*, esc*^*2*^* brm*^*2*^ and *Trl*^*R85*^(Fig. 5C). The survivors from *Psc*^*1*^ and *esc*^*2*^ were quite healthy as compared to the survivors of *Cg-Gal4* > *UAS abd-A.* This reduction in lethality indicates that Esc and Psc proteins are strongly suppressing the melanotic pseudo-tumor phenotype and its consequences on development. Although *brm*^*2*^ showed an increase in penetrance it decreases pupal lethality 89% compare to *abd-A* alone. *Trl*^*R85*^ showed a decrease in pupal lethality (79%) (Fig. 5C). While designing the study, we hypothesized that the observed leukemia like phenotype was due to the aberrant transcription of genes that led to the misregulation of hematopoiesis. It followed therefore that mutations in genes that maintained repression would result in an enhancement of the phenotype (PcG) whereas mutations in genes that maintained transcriptionally states of genes would have the opposite effect (trxG). Our results do not reflect such a clear-cut enhancement or suppression of the phenotype.

### Relative strength of the Gal4 drivers

**Figure 6:**
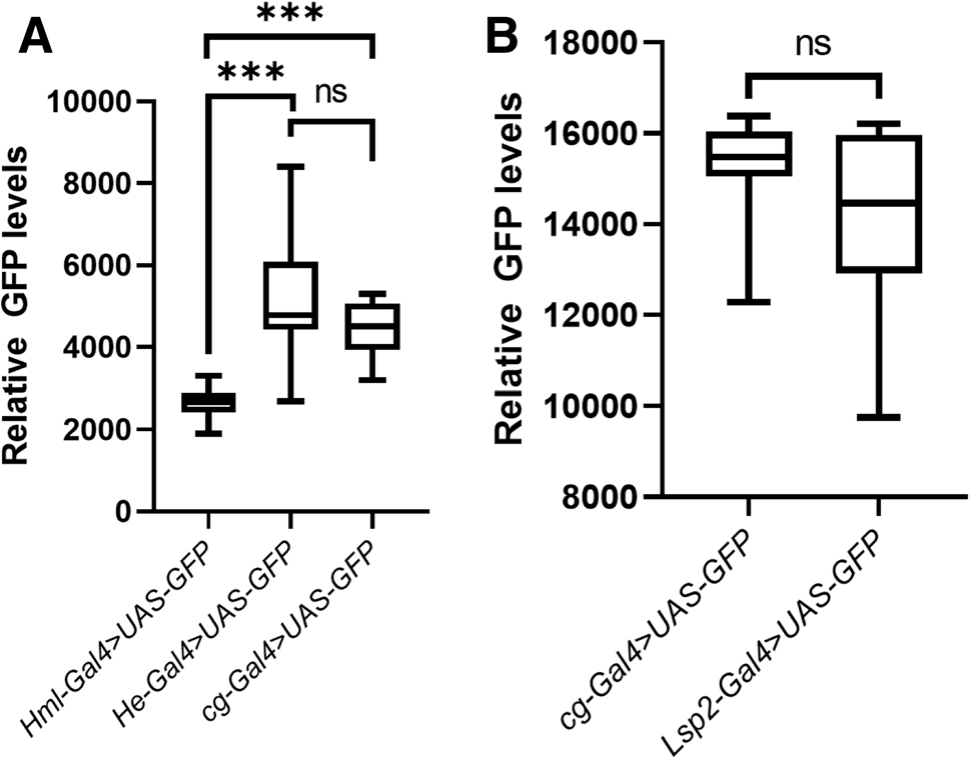
Quantification of relative Gal4 strength by relative GFP levels. *HmlD3-Gal4, He Gal4, cg-Gal4* and *Lsp2-Gal4* were used to drive the expression of *UAS-mcd8-GFP.* (**A**) Average maximum GFP intensities of whole larvae of *HmlD3-Gal4* > *UAS mcd8-GFP* and *He-Gal4* > *UAS mcd8-GFP* were compared with regions excluding the fat body in *Cg-Gal4* > *UAS mcd8-GFP.* The *HmlD3-Gal4* driver was weaker than *He-Gal4* (*p* < 0.0001) as well as *Cg-Gal4* (*p* = 0.0005). *He-Gal4* was not significantly different from *Cg-Gal4* (*p* = 0.1903) (**B**) Average maximum intensities of whole larvae of *Cg-Gal4* > *UAS mcd8-GFP* were compared with *Lsp2-Gal4* > *UAS-GFP*. GFP levels were not significantly different (*p* = .1261).

One possibility that may explain the difference in the penetrance of our melanotic pseudo tumor phenotype is that the relative expression levels of the genes differ significantly under different Gal4 drivers. To test the relative strength of the Gal4s, we overexpressed mcd8-GFP under *Cg-Gal4, HmlD3-Gal4, He-Gal4* and *Lsp2-Gal4. HmlD3-Gal4* was significantly weaker than *He-Gal4* and *cg-Gal4. He-Gal4* and *Cg-Gal4* appear to drive expression at similar levels. However, as we compared whole *He-GFP* expressing larvae to regions devoid of the fatbody in C*g-Gal4* larvae, this similarity may be artefactual (Fig. 6A, Supplementary Table 11A). *Lsp2-Gal4* and *Cg-Gal4* drove GFP at similar levels in the fatbody (Fig. 6B, Supplementary Table S11B). of the respective drivers in hemocytes (Figs. 1 and 2A,B and Supplementary Table S3). Taken together, this implied that the melanized pseudo-tumour phenotype we observe is of hemocyte origin. However, as we do see a lower but significant increase in blood cells when Lsp2-Gal4 is used, as well as many lamellocytes in *Lsp2-Gal4* > *Ubx*, the difference in the phenotypic penetrance when using blood specific divers compared to *Cg-Gal4* may be due to non-cell autonomous cues from the fatbody^80^.

### Transcriptomic analysis of abd-A overexpression

Blood from the *Cg-Gal4* > *UAS abd-A* larvae and *Cg-Gal4* > *CS* larvae were extracted and subjected to RNA-Seq analysis. The raw RNAseq data is available at the NCBI GEO database under the accession number GSE163983. The sequenced reads were mapped to the fruit fly genome (dm6) with an alignment efficiency of ~ 80%. Read counts for the genes were obtained and used for differential expression analysis. The genes were filtered for low counts < 10 which resulted in 11,624 genes. Differentially expressed transcripts were filtered using an adjusted *p* value of < 0.05. Genes were classified as upregulated or downregulated based on twofold change in either direction. This resulted in 444 upregulated (Supplementary Table S13) and 2290 downregulated genes (Supplementary Table S14) in abdA overexpressed larvae when compared to control. Gene ontology enrichment analysis on upregulated and downregulated gene sets reveal distinct biological processes associated with both sets (Supplementary Fig. 4B,C). Among genes upregulated, those involved in response to biotic stimuli, defense response and immune system processes were relatively enriched. Among those downregulated, no such trend was observed. Instead, various biosynthetic processes were broadly downregulated.

*abd-A* was upregulated 44 times the control in *Cg-Gal4* > *UAS abd-A* larvae. *upd2* is upregulated 144 times, and *upd3* is upregulated 66 times in hemocytes that overexpress *abd-A* under *Cg-Gal4*. *upd2 and upd3* are cytokines that induce JAK/STAT signaling via the receptor domless^81^. *upd2* and *upd3* have been implicated in hemocyte proliferation in response to tissue damage and tumors^69^, and lamellocyte differentiation in wild type as well as parasitized larvae^82^. *Pvf2* is upregulated 45 times. *Pvf2* overexpression is sufficient to induce hemocyte proliferation^83^. Spätzle-Processing Enzyme is responsible for activating Spätzle, the Toll ligand^84^. As Toll activation does lead to hemocyte proliferation and differentiation^85,86^, increased levels of Spätzle-Processing Enzyme levels may lead to increased Toll activation. Atilla is upregulated about 117 times, and mysopheroid 27 times, which is consistent with our observation that some plasmatocytes appeared mys+. Additionally, the plasmatocyte marker NimC1 was downregulated 0.26 times. *PPO3* was upregulated 10 times, while *PPO1* and *2* were downregulated 0.07 and 0.05 times respectively. PPO1 and 2 are restricted to crystal cells while PPO3 is expressed in lamellocytes^87^, consistent with the observed increase in lamellocyte differentiation. Interestingly, the Hox gene *labial* was downregulated 0.008 times, implying that this Hox gene is natively expressed in hemocytes.

## Discussion

Homeotic genes or Hox genes determine the cell identity across the anterior–posterior body axis early during development, a function that is conserved across all bilaterians. The later functions played by these genes, however, are less well studied. Several reports indicate that they play a variety of non-homeotic functions later in development. Our lab has shown that *abd-A*, one of the three Hox genes of the bithorax complex of *Drosophila,* acts as a growth promoter in Histoblast Nest Cells^88^. We show this to be a normal additional function of *abd-A* which involved adult cuticle formation during pupation. In the present study, we tested four Hox genes of *Drosophila* by ectopically expressing them in the blood cells and show that they are capable of inducing melanized bodies in circulation. These melanotic spots appear only when expressed in the lymph gland and circulating blood cells. The ectopic expression of the Hox genes also triggers cell proliferation. The cells appear to divide in a cell-autonomous manner, which is reflected in the detection of PH3+ cells in circulation. The presence of *myospheroid* positive elongated cells, seen in circulation, also suggests that Hox overexpression leads to the differentiation of the circulating blood cells into lamellocytes. Overexpression of *abd-A* shows a relatively stronger phenotype while *Abd-B* overexpression does not. It supports an earlier finding in which we observed a non-homeotic growth promoter role of *abd-A* but not of *Abd-B* during the formation of the adult cuticle during pupations^88^. However, as the Hox genes were expressed from UAS lines that were not inserted within the same locus, variations in the tumor penetrance that we obtained may be due to differences in the level of expression between the genes as opposed to the leukemogenicity of the individual genes.

The fact that the cells appear to be phenotypically confined to and do not induce cell fates outside of hemocytes, implies that expression of these genes works in tandem with, and above the specific program of the cell types. It is known from previous studies that Hox dysregulation in leukemia is usually concomitant with gain or loss of function mutations in upstream regulators, most commonly in Mixed Lineage Leukemia-1 (MLL-1) fusion proteins^89,90^, or loss of function Enhancer of Zeste Homolog 2 (EZH2) mutations^91^. It has been reported that EZH2 mutations have the lowest number of co-operating mutations to induce leukemogenesis^92^.

Interestingly, we observe polycomb members *Psc* and *Esc* have a role in suppressing melanized pseudo-tumor formation. Both *Psc* and *Esc* mutants rescued the phenotype significantly which suggests that tumor suppressor genes may be their targets for repression. However, while there is evidence that Hox overexpression in vertebrate blood cells do induce leukemia, the modulation of the phenotype by PcG and trxG mutant backgrounds may be due to the differential regulation of immune genes in the overexpression background. As many Hox induced leukemias occur in the background of PcG loss of function and trxG gain of function backgrounds, this may lead to the differential accessibility of the overexpressed transcription factor to immune genes, thus either enhancing or suppressing the phenotype. While PcG and trxG genes are known to function in a complex, results in our lab indicate that they may have functions outside their canonical pathways, which would explain why genes within similar complexes elicit different effects on our overexpression backgrounds^93,94^. However, cannot discount the possibility that the nature of the alleles used in the study contribute to enhancement or suppression displayed in our results, especially since we have not used multiple alleles in each experiment. Taken together, we speculate that Hox gene activation in hemocytes causes cell-autonomous proliferation and differentiation and induces leukemia via aberrant transcription.

Our RNAseq data shows significant upregulation of key signaling proteins. upd2 and 3 levels are involved in hemocyte proliferation in existing tumor models^69^ as well as lamellocyte differentiation in response to parasitoid wasp infection^82^. Aberrant JAK2 signaling has been implicated in vertebrate leukemias^14^. Thus, it is likely that the increased transcription of these two cytokines may be causal to our observed phenotype. Pvf2 is another signaling factor, which when overexpressed leads to hemocyte proliferation^83^. VEGF-C, the human homologue of Pvf2, is involved in signaling implicated in myeloid leukemias^95^. We also observed the upregulation of Spätzle-Processing Enzyme (SPE), the enzyme responsible for activating the Toll ligand Spätzle. Increased levels of SPE may increase Toll signaling within the hemocytes. It has already been demonstrated that constitutively active Toll can lead to hemocyte proliferation and differentiation^86^. Thus, taken together, it is likely that hemocyte proliferation and differentiation induced by abd-A overexpression may be via signaling events involving these genes.

Our results indicate that Hox genes are causal in leukemia, reinforcing previous studies in vertebrate model systems, and extending these findings to *Drosophila*. This also opens the possibility that Hox gene induced leukemias, especially those of the myeloid lineage, can be studied and modelled in *Drosophila,* as the hallmarks of previously studied leukemias are observed, i.e., the increased number of hemocytes, circulating lamellocytes (including plasmatocytes differentiating into lamellocytes) and melanized pseudo tumors. Till date the only known Hox genes to participate in *Drosophila* hematopoiesis are *Antp* which marks in the PSC^43^, and *Ubx* which provides spatial signals for the development of the LG^45^. In vertebrates, Hox genes have been shown to express within progenitor cells and are rapidly switched off during cell maturation. As our overexpression lines perturb both cell number and differentiation, it is possible that multiple *Drosophila* Hox genes are involved in finetuning the precise program of *Drosophila* blood cell development as well. abd-A overexpression leads to the downregulation of labial (Supplementary Table S14), which implies that labial is natively transcribed in some, if not all hemocytes. What role it plays in normal hematopoiesis remains to be studied.

In summary, *Drosophila* the Hox genes *Dfd, Ubx and* particularly *abd-A*, when expressed in blood cells, are leukemogenic. This link of Hox genes to the pseudo-tumor phenotype supports the non-homeotic role of *abd-A* as a growth promoter later during development. The disease phenotype is modified by select PcG/trxG members. This reinforces previous studies in vertebrates that report the mis-regulation of Hox genes in several cancers and implicate epigenetic factors in them. Studying hox induced leukemias in *Drosophila* offers advantages of the fly model in exploring the biology of leukemogenesis to develop novel potential markers and therapeutic options, some of which may be represented within our RNAseq data.

## Materials and methods

### Fly strains and culture

Flies were cultured in standard cornmeal and sucrose agar. The wild-type flies used in this study were Canton-S. Flies were maintained at 25 °C. For all experiments, flies laid eggs for 6 h before being transferred to a fresh vial. Larvae were screened and used for immunohistochemistry at 96–102 h post egg laying, before the onset of metamorphosis. Supplementary Tables S1 and S2 list the fly stocks used in this study.

### Larval screening for percent penetrance and severity of the phenotype

For the over-expression of different Hox genes, the UAS-Gal4 binary system was used. To assess the effect of PcG and trxG members had in modifying the phenotype, heterozygous mutant lines were recombined with the *Cg-Gal4* driver (Supplementary Table S2 for all recombined stocks made in the lab). Confirmation of recombination was based on expression of *w*+ linked with the *Cg-Gal4* transgene and lethality when backcrossed with the mutant line. Recombined mutants with *Cg-Gal4* were maintained over the *CyO-GFP* balancer for GFP screening. Third chromosome mutants were crossed with homozygous *Cg-Gal4* lines and maintained over TM6B for screening via the *Tubby* phenotype. Experimental crosses were set between recombined strains (*Cg-Gal4* with mutant) and *UAS-abd-A* at a density of 12 females and 6 males for each cross. Egg lay was allowed for 6 h and progeny were collected after 96 h post egg lay, at the L3F stage. Screening was done using a stereomicroscope. Penetrance was calculated by calculating the percentage of melanotic pseudo-tumor manifesting larvae. Severity of the phenotype was assessed visually. One-way ANOVA (Dunnett's multiple comparisons) was performed to test the significance.

### Pupal lethality count

To assess the pupal lethality, larvae were allowed to develop into pupae and were observed beyond 10 days post egg lay. Eclosed progenies were considered as survivors. Dead pupae were counted manually.

For heterozygous mutant experimental pupae, larvae were first screened to confirm the presence of the *Cg-Gal4* driven expression of *UAS-abd-A* and the presence of the mutation before being transferred to fresh vials. Second chromosome mutants were confirmed by selecting non-GFP larvae while third chromosome mutations were *Tb*^+^.

### Immunostaining and cell quantification

For staining proliferative cells we made use the M -phase marker, Anti-Phosphohistone 3 at serine 10, from Upstate (cat# 07-212, 1 ng/μL). For confirming the presence of lamellocytes, we used anti-myospheroid (DSHB #CF.6G11, 27 pg/μL). Blood cells were prepared using an established protocol^65^. Blood cells numbers were quantified using a modified version of the protocol by Petraki et al.^64^. Larvae were dissected in 4 mm wells, their hemolymph allowed to settle down, before being fixed with 1% formaldehyde and stained with DAPI. Each well was scanned using an Olympus IX83 at 20X, with 32 images stitched. Cells were quantified using CellProfiler by counting individual nuclei. Significance was tested using an unpaired t-test with Welch's correction between control and overexpression genotypes.

### Visualization of lymph glands in larvae over expressing Hox genes and Quantification of relative GFP levels

Larvae were grown as described above. Virgin *HmlD3-Gal4, UAS-GFP,* flies were used to drive the expression of the individual Hox genes. *Cg-Gal4, HmlD3-Gal4, He-Gal4* and *Lsp2-Gal4* lines were crossed with *UAS-mcd8*-*GFP* lines Larvae were harvested and visualized under a Zeiss Axiozoom.V16 for GFP. For comparison between *He-Gal4, HmlD3-Gal4* and *cg-Gal4,* whole larval maximum intensities of *He-Gal4* and *HmlD3-Gal4* were compared with regions devoid of the fatbody in *cg-Gal4*. For comparison between ***cg-Gal4*** and *Lsp2-Gal4,* hole larval maximum intensities were compared.

### RNA isolation, sequencing and data processing

RNA profiles were compared between Cg-Gal4 > CS and Cg-Gal4 > UAS-abd-A larvae. Larvae were dissected in 4 mm wells with 20 uL PBS. After each dissection, the hemolymph and PBS were transferred into a microfuge tube on ice. The hemolymph of 20 larvae was thus collected, pelleted and resuspended in 20 uL of TRIzol.

500 ng of total RNA was utilized for mRNA isolation, fragmentation and priming. First strand synthesis was carried out in the presence of Actinomycin D followed by second strand synthesis. The obtained double stranded cDNA was purified using magnetic beads, end-repaired, adenylated and ligated to Illumina multiplex barcode adapters as per the NEBNext Ultra Directional RNA Library Prep Kit protocol. The adapter ligated cDNA was purified using magnetic beads and was subjected to 12 cycles of Indexing-PCR with the following conditions: 37 °C for 15mins, denaturation at 98 °C for 30 s, 12 cycles of 98 °C for 10 s and 65 °C for 75 s, followed by a final extension of 65 °C for 5 min to enrich the adapter-ligated fragments. The sequencing library thus obtained by PCR was purified with magnetic beads, followed by library quality control checks. The sequencing library was quantified by Qubit fluorometer. An Agilent 2200 TapeStation was used to analyze fragment size distribution. Finally, Kapa Library Quantification Kit was used to quantify the sequencing library by quantitative PCR. Whole genome RNAseq was performed on Illumina NextSeq 500 to obtain paired end libraries of read length 75 X 2 with at least 25 million reads per sample. The raw RNAseq data is available at the NCBI GEO database under the accession number GSE163983.

Raw data obtained from blood tissue was processed using FastQC^96^ to assess sequence quality. The sequence reads were mapped against the fruit fly reference genome (dm6) using the aligner STAR^97^. Read counts were calculated using the htseq-count module of HTSeq^98^. Differential expression was detected between the control and abdA overexpressed samples based on a negative binomial generalized linear model and Wald test for significance testing using DESeq2^99^. Transcripts were deemed as differentially expressed if they crossed a two-fold change (FC) threshold; i.e. upregulated transcripts (F.C. >  = 2) and downregulated transcripts (F.C. < 0.5). Genes identified as differentially regulated were processed for gene ontology enrichment analyses using the enrichGO module of clusterProfiler^100^. A q-value cutoff of 0.05 was used to filter significant biological processes and visualized along with the number of contributing genes.

### Ethical approval and consent for publication

All the authors have consented for publication of this work.

## Supplementary Information

Supplementary Information 1.

Supplementary Information 2.

Supplementary Information 3.

Supplementary Information 4.

## Data Availability

Data and material are available on request.

## Abbreviations

PH3: Phospho histone 3 at serine 10
LG: Lymph gland
PcG: Polycomb group of proteins
TrxG: Trithorax group of proteins
PRC1: Polycomb repressive complex 1
PRC2: Polycomb repressive complex 2
