# Supplementary material for: Drosophila Hox genes induce melanized pseudo-tumors when misexpressed in hemocytes
Source: Sci Rep. 2021 Jan 19;11:1838. doi: 10.1038/s41598-021-81472-5 (PMC7815749; doi:10.1038/s41598-021-81472-5)
Supplement: Supplementary file 1 — Supplementary Information 1. [file 41598_2021_81472_MOESM1_ESM.docx]

**Supplementary Table 1:** List of fly strains used in the study

| **Fly strains** | **Description** |
| --- | --- |
| *Pc^1^* | Polycomb, amorphic allele, obtained from Francois Karch lab |
| *Psc^1^* | Posterior sex comb, hypomorphic allele, obtained from Francois Karch lab |
| *Su(z)2^1.a1^* | supressor of zeste, loss of function allele, BDSC |
| *E(z)^731^* | Enhancer of zeste,EMS mutation, BDSC |
| *esc^2^* | extra sex comb,amorphic allele, obtained from Francois Karch lab |
| *Su(z)12^3^* | Supressor of zeste 12,EMS mutation, BDSC |
| *Trl^R85^* | trithorax-like,amorphic allele, obtained from Francois Karch lab |
| *brm2* | brahma 2,amorphic allele, BDSC |
| *Cg-Gal4* | hemocytes and fat body specific driver, BDSC |
| *He-Gal4* | hemocytes specific driver, BDSC |
| *HmlD3-Gal4* | hemocytes specific driver, BDSC |
| *Lsp2-Gal4* | Fat body specific driver, BDSC |
| *UAS-Dfd-HA* | Yacine Graba's Lab |
| *UAS-Ubx-HA* | Yacine Graba's Lab |
| *UAS-abd-A-HA* | Yacine Graba's Lab |
| *UAS-Abd-B-HA* | Yacine Graba's Lab |

**Supplementary Table 2:** List of Gal4 flies stocks in PcG mutant background

| **Stocks** | **Description** |
| --- | --- |
| *Cg-Gal4; Pc^1^/TM6B* | combination of Cg-Gal 4 transgene and Pc^1^ |
| *Cg-Gal4, Psc^1^/CyO-GFP* | recombination between Cg-Gal 4 and Psc^1^ |
| *Cg-Gal4, Su(z)2^1.a1^/CyO-GFP* | recombination between Cg-Gal 4 and Su(z)2^1.a1^ |
| *Cg-Gal4; E(z)^731^/TM6B* | combination of Cg-Gal 4 transgene and E(z)^731^ |
| *Cg-Gal4, esc^2^/CyO-GFP* | recombination between Cg-Gal 4 and esc^2^ |
| *Cg-Gal4; Su(z) 12^3^red^1^e^1^/TM6B* | combination of Cg-Gal 4 transgene and *Su(z) 12^3^* |
| *Cg-Gal4; Trl^R85^/TM6B* | combination of Cg-Gal 4 transgene and *Trl^R85^* |
| *Cg-Gal4; brm^2^/TM6B* | combination of Cg-Gal 4 transgene and *brm^2^* |

| **Genotype** | **Experiment** | **Larvae screened** | **Larvae with tumor** | **Percentage** | **Average** | **Std. Dev.** |
| --- | --- | --- | --- | --- | --- | --- |
| Cg-Gal4 | 1 | 50 | 0 | 0.00 | 0.00 | 0 |
|  | 2 | 50 | 0 | 0.00 |  |  |
|  | 3 | 50 | 0 | 0.00 |  |  |
| Cg-GAL4 > UAS-Dfd | 1 | 50 | 13 | 26.00 | 26.67 | 7.02 |
|  | 2 | 50 | 10 | 20.00 |  |  |
|  | 3 | 50 | 17 | 34.00 |  |  |
| Cg-GAL4 > UAS-Ubx | 1 | 50 | 20 | 40.00 | 40.00 | 4 |
|  | 2 | 50 | 22 | 44.00 |  |  |
|  | 3 | 50 | 18 | 36.00 |  |  |
| Cg-GAL4 > UAS-abd-A | 1 | 106 | 79 | 74.53 | 82.10 | 6.56 |
|  | 2 | 112 | 96 | 85.71 |  |  |
|  | 3 | 122 | 105 | 86.07 |  |  |
| Cg-GAL4 > UAS-Abd-B | 1 | 50 | 2 | 4.00 | 2.67 | 1.15 |
|  | 2 | 50 | 1 | 2.00 |  |  |
|  | 3 | 50 | 1 | 2.00 |  |  |
| Lsp2-Gal4 | 1 | 50 | 0 | 0.00 | 0.00 | 0 |
|  | 2 | 50 | 0 | 0.00 |  |  |
|  | 3 | 50 | 0 | 0.00 |  |  |
| Lsp2-Gal4 > UAS-Dfd | 1 | 50 | 0 | 0.00 | 0.00 | 0 |
|  | 2 | 50 | 0 | 0.00 |  |  |
|  | 3 | 50 | 0 | 0.00 |  |  |
| Lsp2-Gal4 > UAS-Ubx | 1 | 50 | 0 | 0.00 | 0.00 | 0 |
|  | 2 | 50 | 0 | 0.00 |  |  |
|  | 3 | 50 | 0 | 0.00 |  |  |
| Lsp2-Gal4 > UAS-abd-A | 1 | 50 | 0 | 0.00 | 0.00 | 0 |
|  | 2 | 50 | 0 | 0.00 |  |  |
|  | 3 | 50 | 0 | 0.00 |  |  |
| Lsp2-Gal4 > UAS-Abd-B | 1 | 50 | 0 | 0.00 | 0.00 | 0 |
|  | 2 | 50 | 0 | 0.00 |  |  |
|  | 3 | 50 | 0 | 0.00 |  |  |
| HmlD3-Gal4 | 1 | 50 | 0 | 0.00 | 0.00 | 0 |
|  | 2 | 50 | 0 | 0.00 |  |  |
|  | 3 | 50 | 0 | 0.00 |  |  |
| HmlD3-Gal4 > UAS-Dfd | 1 | 50 | 4 | 8.00 | 6.00 | 2 |
|  | 2 | 50 | 2 | 4.00 |  |  |
|  | 3 | 50 | 3 | 6.00 |  |  |
| HmlD3-Gal4 > UAS-Ubx | 1 | 50 | 2 | 4.00 | 2.67 | 2.30 |
|  | 2 | 50 | 0 | 0.00 |  |  |
|  | 3 | 50 | 2 | 4.00 |  |  |
| HmlD3-Gal4 > UAS-abd-A | 1 | 50 | 2 | 4.00 | 4.00 | 2 |
|  | 2 | 50 | 3 | 6.00 |  |  |
|  | 3 | 50 | 1 | 2.00 |  |  |
| HmlD3-Gal4 > UAS-Abd-B | 1 | 50 | 1 | 2.00 | 2.67 | 1.15 |
|  | 2 | 50 | 1 | 2.00 |  |  |
|  | 3 | 50 | 2 | 4.00 |  |  |
| He-Gal4 | 1 | 50 | 0 | 0.00 | 0.00 | 0 |
|  | 2 | 50 | 0 | 0.00 |  |  |
|  | 3 | 50 | 0 | 0.00 |  |  |
| He-Gal4 > UAS-Dfd | 1 | 50 | 2 | 4.00 | 3.33 | 1.15 |
|  | 2 | 50 | 2 | 4.00 |  |  |
|  | 3 | 50 | 1 | 2.00 |  |  |
| He-Gal4 > UAS-Ubx | 1 | 50 | 6 | 12.00 | 9.33 | 2.31 |
|  | 2 | 50 | 4 | 8.00 |  |  |
|  | 3 | 50 | 4 | 8.00 |  |  |
| He-Gal4 > UAS-abd-A | 1 | 50 | 5 | 10.00 | 8.00 | 2 |
|  | 2 | 50 | 4 | 8.00 |  |  |
|  | 3 | 50 | 3 | 6.00 |  |  |
| He-Gal4 > UAS-Abd-B | 1 | 50 | 2 | 4.00 | 3.33 | 1.15 |
|  | 2 | 50 | 2 | 4.00 |  |  |
|  | 3 | 50 | 1 | 2.00 |  |  |

**Supplementary Table 3:** Tumor phenotype in Hox gene over-expressed larvae

| **Genotype** | **Experiment** | **Total pupae** | **Dead pupae** | **Lethality (%)** | **Average** | **Std. Dev.** |
| --- | --- | --- | --- | --- | --- | --- |
| Cg-Gal4 | 1 | 263 | 1 | 0.38 | 0.13 | 0.22 |
|  | 2 | 206 | 0 | 0.00 |  |  |
|  | 3 | 201 | 0 | 0.00 |  |  |
| Cg-GAL4 > UAS-Dfd | 1 | 262 | 145 | 55.34 | 52.62 | 3.04 |
|  | 2 | 252 | 134 | 53.17 |  |  |
|  | 3 | 225 | 111 | 49.33 |  |  |
| Cg-GAL4 > UAS-Ubx | 1 | 335 | 85 | 25.37 | 23.81 | 5.06 |
|  | 2 | 215 | 60 | 27.91 |  |  |
|  | 3 | 237 | 43 | 18.14 |  |  |
| Cg-GAL4 > UAS-abd-A | 1 | 86 | 84 | 97.67 | 99 | 1.34 |
|  | 2 | 96 | 96 | 100.00 |  |  |
|  | 3 | 80 | 80 | 100.00 |  |  |
| Cg-GAL4 > UAS-Abd-B | 1 | 274 | 6 | 2.19 | 1.62 | 1.42 |
|  | 2 | 150 | 4 | 2.67 |  |  |
|  | 3 | 151 | 0 | 0.00 |  |  |
| Lsp2-Gal4 | 1 | 220 | 0 | 0.00 | 0.21 | 0.36 |
|  | 2 | 159 | 1 | 0.63 |  |  |
|  | 3 | 189 | 0 | 0.00 |  |  |
| Lsp2-Gal4 > UAS-Dfd | 1 | 151 | 12 | 7.95 | 9.04 | 4.80 |
|  | 2 | 154 | 22 | 14.29 |  |  |
|  | 3 | 164 | 8 | 4.88 |  |  |
| Lsp2-Gal4 > UAS-Ubx | 1 | 198 | 68 | 34.34 | 26.07 | 7.18 |
|  | 2 | 131 | 28 | 21.37 |  |  |
|  | 3 | 160 | 36 | 22.50 |  |  |
| Lsp2-Gal4 > UAS-abd-A | 1 | 222 | 100 | 45.05 | 30.60 | 13.55 |
|  | 2 | 182 | 52 | 28.57 |  |  |
|  | 3 | 253 | 46 | 18.18 |  |  |
| Lsp2-Gal4 > UAS-Abd-B | 1 | 211 | 1 | 0.47 | 0.16 | 0.27 |
|  | 2 | 166 | 0 | 0.00 |  |  |
|  | 3 | 204 | 0 | 0.00 |  |  |
| HmlD3-Gal4 | 1 | 97 | 1 | 1.03 | 0.34 | 0.60 |
|  | 2 | 117 | 0 | 0.00 |  |  |
|  | 3 | 95 | 0 | 0.00 |  |  |
| HmlD3-Gal4 > UAS-Dfd | 1 | 49 | 1 | 2.04 | 6.10 | 3.54 |
|  | 2 | 90 | 7 | 7.78 |  |  |
|  | 3 | 106 | 9 | 8.49 |  |  |
| HmlD3-Gal4 > UAS-Ubx | 1 | 42 | 1 | 2.38 | 1.80 | 1.60 |
|  | 2 | 44 | 0 | 0.00 |  |  |
|  | 3 | 66 | 2 | 3.03 |  |  |
| HmlD3-Gal4 > UAS-abd-A | 1 | 45 | 2 | 4.44 | 3.00 | 2.60 |
|  | 2 | 88 | 4 | 4.55 |  |  |
|  | 3 | 56 | 0 | 0.00 |  |  |
| HmlD3-Gal4 > UAS-Abd-B | 1 | 51 | 0 | 0.00 | 0.00 | 0 |
|  | 2 | 53 | 0 | 0.00 |  |  |
|  | 3 | 31 | 0 | 0.00 |  |  |
| He-Gal4 | 1 | 136 | 0 | 0.00 | 1.23 | 1.59 |
|  | 2 | 165 | 5 | 3.03 |  |  |
|  | 3 | 149 | 1 | 0.67 |  |  |
| He-Gal4 > UAS-Dfd | 1 | 99 | 8 | 8.08 | 3.92 | 3.78 |
|  | 2 | 135 | 4 | 2.96 |  |  |
|  | 3 | 141 | 1 | 0.71 |  |  |
| He-Gal4 > UAS-Ubx | 1 | 135 | 14 | 10.37 | 8.82 | 2.02 |
|  | 2 | 136 | 13 | 9.56 |  |  |
|  | 3 | 153 | 10 | 6.54 |  |  |
| He-Gal4 > UAS-abd-A | 1 | 86 | 11 | 12.79 | 12.19 | 0.57 |
|  | 2 | 140 | 17 | 12.14 |  |  |
|  | 3 | 103 | 12 | 11.65 |  |  |
| He-Gal4 > UAS-Abd-B | 1 | 139 | 2 | 1.44 | 1.76 | 1.37 |
|  | 2 | 184 | 6 | 3.26 |  |  |
|  | 3 | 173 | 1 | 0.58 |  |  |

**Supplementary Table 4:** Pupal lethality in Hox genes over-expression by different drivers

**Supplementary Table 5:** Lamellocytes count in Cg-Gal4 driven Hox genes over-expressed larvae

| **Genotype** | **Total blood cells count/larva** | **Lamellocytes** | **percentage** | **Avg. percentage** | **Std. Dev.** |
| --- | --- | --- | --- | --- | --- |
| Cg-Gal4 | 3112 | 1 | 0.03 | 0.05 | 0.05 |
|  | 5053 | 3 | 0.06 |  |  |
|  | 3490 | 3 | 0.09 |  |  |
|  | 4072 | 2 | 0.05 |  |  |
|  | 3652 | 0 | 0.00 |  |  |
|  | 3625 | 1 | 0.03 |  |  |
|  | 4441 | 0 | 0.00 |  |  |
|  | 2400 | 3 | 0.13 |  |  |
|  | 6645 | 5 | 0.08 |  |  |
|  | 4456 | 0 | 0.00 |  |  |
|  | 3402 | 1 | 0.03 |  |  |
|  | 5407 | 7 | 0.13 |  |  |
| Cg-Gal4 > UAS-Dfd | 9295 | 73 | 0.79 | 0.74 | 0.45 |
|  | 4376 | 84 | 1.92 |  |  |
|  | 8905 | 38 | 0.43 |  |  |
|  | 11696 | 39 | 0.33 |  |  |
|  | 11793 | 68 | 0.58 |  |  |
|  | 7854 | 64 | 0.81 |  |  |
|  | 6238 | 66 | 1.06 |  |  |
|  | 14974 | 32 | 0.21 |  |  |
|  | 6949 | 31 | 0.45 |  |  |
|  | 6069 | 58 | 0.96 |  |  |
|  | 5596 | 40 | 0.71 |  |  |
|  | 9639 | 60 | 0.62 |  |  |
| Cg-Gal4 > UAS-Ubx | 13731 | 115 | 0.84 | 2.79 | 1.73 |
|  | 2150 | 130 | 6.05 |  |  |
|  | 3611 | 180 | 4.98 |  |  |
|  | 7503 | 96 | 1.28 |  |  |
|  | 6356 | 38 | 0.60 |  |  |
|  | 5310 | 150 | 2.82 |  |  |
|  | 4640 | 62 | 1.34 |  |  |
|  | 4219 | 170 | 4.03 |  |  |
|  | 2511 | 90 | 3.58 |  |  |
|  | 3978 | 130 | 3.27 |  |  |
|  | 3099 | 43 | 1.39 |  |  |
|  | 8261 | 270 | 3.27 |  |  |
| Cg-Gal4 > UAS-abd-A | 6823 | 211 | 3.09 | 4.45 | 1.05 |
|  | 8404 | 334 | 3.97 |  |  |
|  | 8928 | 394 | 4.41 |  |  |
|  | 9504 | 450 | 4.73 |  |  |
|  | 14260 | 465 | 3.26 |  |  |
|  | 17011 | 538 | 3.16 |  |  |
|  | 12775 | 680 | 5.32 |  |  |
|  | 14838 | 730 | 4.92 |  |  |
|  | 14672 | 670 | 4.57 |  |  |
|  | 15547 | 710 | 4.57 |  |  |
|  | 16363 | 750 | 4.58 |  |  |
|  | 9190 | 630 | 6.86 |  |  |
| Cg-Gal4 > UAS-Abd-B | 6806 | 0 | 0.00 | 0.01 | 0.01 |
|  | 6481 | 0 | 0.00 |  |  |
|  | 10413 | 3 | 0.03 |  |  |
|  | 6414 | 0 | 0.00 |  |  |
|  | 7085 | 0 | 0.00 |  |  |
|  | 11030 | 0 | 0.00 |  |  |
|  | 7414 | 0 | 0.00 |  |  |
|  | 12780 | 0 | 0.00 |  |  |
|  | 5776 | 2 | 0.03 |  |  |
|  | 9093 | 1 | 0.01 |  |  |
|  | 7957 | 0 | 0.00 |  |  |

**Supplementary Table 6:** Lamellocytes count in He-Gal4 driven Hox genes over-expressed larvae

| **Genotype** | **Total blood cells count/larva** | **Lamellocytes** | **percentage** | **Avg. percentage** | **Std. Dev.** |
| --- | --- | --- | --- | --- | --- |
| He-Gal4 | 5043 | 0 | 0.00 | 0.06 | 0.08 |
|  | 915 | 0 | 0.00 |  |  |
|  | 6403 | 9 | 0.14 |  |  |
|  | 6443 | 18 | 0.28 |  |  |
|  | 5322 | 5 | 0.09 |  |  |
|  | 7319 | 1 | 0.01 |  |  |
|  | 2958 | 1 | 0.03 |  |  |
|  | 4907 | 2 | 0.04 |  |  |
|  | 2382 | 1 | 0.04 |  |  |
|  | 4241 | 0 | 0.00 |  |  |
|  | 2734 | 1 | 0.04 |  |  |
|  | 1197 | 0 | 0.00 |  |  |
| He-Gal4 > UAS-Dfd | 15120 | 118 | 7.86 | 1.07 | 2.15 |
|  | 10272 | 64 | 0.62 |  |  |
|  | 9361 | 21 | 0.22 |  |  |
|  | 9880 | 58 | 0.59 |  |  |
|  | 12057 | 41 | 0.34 |  |  |
|  | 22549 | 84 | 0.37 |  |  |
|  | 10946 | 13 | 0.12 |  |  |
|  | 15137 | 94 | 0.62 |  |  |
|  | 15325 | 46 | 0.30 |  |  |
|  | 15649 | 70 | 0.45 |  |  |
|  | 11039 | 50 | 0.45 |  |  |
|  | 15901 | 139 | 0.87 |  |  |
| He-Gal4 > UAS-Ubx | 2510 | 109 | 4.34 | 1.66 | 1.337178 |
|  | 6106 | 60 | 0.98 |  |  |
|  | 8210 | 131 | 1.60 |  |  |
|  | 9144 | 55 | 0.60 |  |  |
|  | 6008 | 30 | 0.50 |  |  |
|  | 3993 | 110 | 2.75 |  |  |
|  | 4303 | 140 | 3.25 |  |  |
|  | 11360 | 130 | 1.14 |  |  |
|  | 3935 | 39 | 0.99 |  |  |
|  | 7217 | 31 | 0.43 |  |  |
| He-Gal4 > UAS-abd-A | 3896 | 80 | 2.05 | 2.52 | 2.151202 |
|  | 10428 | 74 | 0.71 |  |  |
|  | 4354 | 90 | 2.07 |  |  |
|  | 8239 | 130 | 1.58 |  |  |
|  | 7003 | 340 | 4.86 |  |  |
|  | 14923 | 31 | 0.21 |  |  |
|  | 11963 | 470 | 3.93 |  |  |
|  | 8448 | 600 | 7.10 |  |  |
|  | 10826 | 210 | 1.94 |  |  |
|  | 8206 | 66 | 0.80 |  |  |
| He-Gal4 > UAS-Abd-B | 2760 | 0 | 0.00 | 0.01 | 0.023719 |
|  | 5471 | 0 | 0.00 |  |  |
|  | 4373 | 0 | 0.00 |  |  |
|  | 4216 | 0 | 0.00 |  |  |
|  | 2752 | 2 | 0.07 |  |  |
|  | 3959 | 0 | 0.00 |  |  |
|  | 5229 | 2 | 0.04 |  |  |
|  | 7314 | 0 | 0.00 |  |  |
|  | 1720 | 0 | 0.00 |  |  |
|  | 5682 | 0 | 0.00 |  |  |
|  | 4266 | 0 | 0.00 |  |  |

**Supplementary Table 7:** Lamellocytes count in HmlD3-Gal4 driven Hox genes over-expressed larvae

| **Genotype** | **Total blood cells count/larva** | **Lamellocytes** | **percentage** | **Avg. percentage** | **Std. Dev.** |
| --- | --- | --- | --- | --- | --- |
| HmlD3-Gal4 | 5452 | 2 | 0.04 | 0.10 | 0.12 |
|  | 4121 | 7 | 0.17 |  |  |
|  | 3651 | 6 | 0.16 |  |  |
|  | 5595 | 1 | 0.02 |  |  |
|  | 4192 | 0 | 0.00 |  |  |
|  | 1888 | 0 | 0.00 |  |  |
|  | 6962 | 1 | 0.01 |  |  |
|  | 4031 | 4 | 0.10 |  |  |
|  | 2811 | 3 | 0.11 |  |  |
|  | 4042 | 5 | 0.12 |  |  |
|  | 4704 | 0 | 0.00 |  |  |
|  | 2143 | 9 | 0.42 |  |  |
| HmlD3-Gal4 > UAS-Dfd | 15925 | 88 | 0.55 | 0.61 | 0.85 |
|  | 7764 | 55 | 0.71 |  |  |
|  | 15067 | 16 | 0.11 |  |  |
|  | 19170 | 44 | 0.23 |  |  |
|  | 17036 | 36 | 0.21 |  |  |
|  | 21543 | 62 | 0.29 |  |  |
|  | 11378 | 10 | 0.09 |  |  |
|  | 19066 | 100 | 0.52 |  |  |
|  | 10120 | 46 | 0.45 |  |  |
|  | 15832 | 84 | 0.53 |  |  |
|  | 14700 | 50 | 0.34 |  |  |
|  | 3556 | 115 | 3.23 |  |  |
| HmlD3-Gal4 > UAS-Ubx | 12673 | 112 | 0.88 | 2.51 | 4.67 |
|  | 10873 | 1 | 0.01 |  |  |
|  | 8090 | 20 | 0.25 |  |  |
|  | 1900 | 310 | 16.32 |  |  |
|  | 13839 | 15 | 0.11 |  |  |
|  | 4096 | 215 | 5.25 |  |  |
|  | 9230 | 6 | 0.07 |  |  |
|  | 12459 | 43 | 0.35 |  |  |
|  | 8312 | 190 | 2.29 |  |  |
|  | 8242 | 320 | 3.88 |  |  |
|  | 6566 | 33 | 0.50 |  |  |
|  | 6883 | 15 | 0.22 |  |  |
| HmlD3-Gal4 > UAS-abd-A | 11169 | 0 | 0.00 | 0.79 | 2.40 |
|  | 13263 | 5 | 0.04 |  |  |
|  | 8060 | 3 | 0.04 |  |  |
|  | 7786 | 7 | 0.09 |  |  |
|  | 2620 | 210 | 8.02 |  |  |
|  | 13229 | 0 | 0.00 |  |  |
|  | 8883 | 12 | 0.14 |  |  |
|  | 10790 | 0 | 0.00 |  |  |
|  | 11701 | 35 | 0.30 |  |  |
|  | 6516 | 3 | 0.05 |  |  |
|  | 10811 | 1 | 0.01 |  |  |
| HmlD3-Gal4 > UAS-abd-B | 4355 | 5 | 0.11 | 0.03 | 0.05 |
|  | 3385 | 0 | 0.00 |  |  |
|  | 5909 | 2 | 0.03 |  |  |
|  | 6469 | 0 | 0.00 |  |  |
|  | 3095 | 4 | 0.13 |  |  |
|  | 3266 | 1 | 0.03 |  |  |
|  | 1836 | 0 | 0.00 |  |  |
|  | 6483 | 0 | 0.00 |  |  |
|  | 2585 | 0 | 0.00 |  |  |
|  | 4449 | 0 | 0.00 |  |  |
|  | 3199 | 0 | 0.00 |  |  |

**Supplementary Table 8:** Lamellocytes count in Lsp2-Gal4 driven Hox genes over-expressed larvae

| **Genotype** | **Total blood cells count/larva** | **Lamellocytes** | **percentage** | **Avg. percentage** | **Std. Dev.** |
| --- | --- | --- | --- | --- | --- |
| Lsp2-Gal4 | 1665 | 0 | 0.00 | 0.00 | 0.00 |
|  | 1574 | 0 | 0.00 |  |  |
|  | 2693 | 0 | 0.00 |  |  |
|  | 2465 | 0 | 0.00 |  |  |
|  | 1663 | 0 | 0.00 |  |  |
|  | 1390 | 0 | 0.00 |  |  |
|  | 1491 | 0 | 0.00 |  |  |
|  | 1441 | 0 | 0.00 |  |  |
|  | 2284 | 0 | 0.00 |  |  |
|  | 1047 | 0 | 0.00 |  |  |
|  | 1398 | 0 | 0.00 |  |  |
| Lsp2-Gal4 > UAS-Dfd | 1061 | 0 | 0.00 | 0.00 | 0.00 |
|  | 3652 | 0 | 0.00 |  |  |
|  | 1138 | 0 | 0.00 |  |  |
|  | 1868 | 0 | 0.00 |  |  |
|  | 1021 | 0 | 0.00 |  |  |
|  | 964 | 0 | 0.00 |  |  |
|  | 941 | 0 | 0.00 |  |  |
|  | 2252 | 0 | 0.00 |  |  |
|  | 2576 | 0 | 0.00 |  |  |
|  | 2205 | 0 | 0.00 |  |  |
| Lsp2-Gal4 > UAS-Ubx | 9846 | 29 | 0.29 | 0.62 | 0.61 |
|  | 3651 | 0 | 0.00 |  |  |
|  | 11544 | 122 | 1.06 |  |  |
|  | 6140 | 82 | 1.34 |  |  |
|  | 8413 | 66 | 0.78 |  |  |
|  | 5571 | 90 | 1.62 |  |  |
|  | 9342 | 118 | 1.26 |  |  |
|  | 7103 | 9 | 0.13 |  |  |
|  | 5849 | 52 | 0.89 |  |  |
|  | 9428 | 1 | 0.01 |  |  |
|  | 5091 | 0 | 0.00 |  |  |
|  | 7734 | 2 | 0.03 |  |  |
| Lsp2-Gal4 > UAS-Abd-A | 2497 | 0 | 0.00 | 0.00 | 0.00 |
|  | 3032 | 0 | 0.00 |  |  |
|  | 2220 | 0 | 0.00 |  |  |
|  | 3460 | 0 | 0.00 |  |  |
|  | 1968 | 0 | 0.00 |  |  |
|  | 4579 | 0 | 0.00 |  |  |
|  | 4443 | 0 | 0.00 |  |  |
|  | 3048 | 0 | 0.00 |  |  |
|  | 3759 | 0 | 0.00 |  |  |
|  | 2351 | 0 | 0.00 |  |  |
|  | 1919 | 0 | 0.00 |  |  |
|  | 1384 | 0 | 0.00 |  |  |
| Lsp2-Gal4 > UAS-Abd-B | 1551 | 0 | 0.00 | 0.39 | 1.29 |
|  | 3004 | 0 | 0.00 |  |  |
|  | 1456 | 0 | 0.00 |  |  |
|  | 1165 | 50 | 4.29 |  |  |
|  | 4101 | 0 | 0.00 |  |  |
|  | 4708 | 0 | 0.00 |  |  |
|  | 3532 | 0 | 0.00 |  |  |
|  | 3429 | 0 | 0.00 |  |  |
|  | 3569 | 0 | 0.00 |  |  |
|  | 3997 | 0 | 0.00 |  |  |
|  | 1087 | 0 | 0.00 |  |  |

| **Genotype** | **Experiment** | | **Larvae screened** | **Larvae with tumour** | **Penetrance (%)** | **Avg.** | **Std. Dev.** |
| --- | --- | --- | --- | --- | --- | --- | --- |
| Cg-Gal4/+ | 1 | 235 | | 0 | 0 | 0 | 0.00 |
|  | 2 | 224 | | 0 | 0 |  |  |
|  | 3 | 222 | | 0 | 0 |  |  |
| Cg-Gal4>UAS-abd-A | 1 | 114 | | 99 | 86.84 | 86.83 | 2.87 |
|  | 2 | 136 | | 122 | 89.7 |  |  |
|  | 3 | 168 | | 141 | 83.96 |  |  |
| Su(z)2^1.a1^, Cg-Gal4>UAS-abd-A | 1 | 183 | | 145 | 79.23 | 80.18 | 4.57 |
|  | 2 | 175 | | 149 | 85.14 |  |  |
|  | 3 | 151 | | 115 | 76.16 |  |  |
| Psc^1^,Cg-Gal4>UAS-abd-A | 1 | 113 | | 20 | 17.70 | 17.93 | 2.93 |
|  | 2 | 119 | | 18 | 15.13 |  |  |
|  | 3 | 124 | | 26 | 20.97 |  |  |
| esc^2^, Cg-Gal4>UAS-abd-A | 1 | 106 | | 16 | 15.09 | 15.18 | 1.59 |
|  | 2 | 113 | | 19 | 16.81 |  |  |
|  | 3 | 88 | | 12 | 13.63 |  |  |
| Cg-Gal4 ; Pc^1^ >UAS-abd-A | 1 | 79 | | 78 | 98.73 | 97.67 | 0.93 |
|  | 2 | 100 | | 97 | 97.00 |  |  |
|  | 3 | 110 | | 107 | 97.27 |  |  |
| Cg-Gal4 ; Su(z)12^3^e^1^red^1^ >UAS-abd-A | 1 | 60 | | 59 | 98.33 | 99.44 | 0.96 |
|  | 2 | 68 | | 68 | 100.00 |  |  |
|  | 3 | 64 | | 64 | 100.00 |  |  |
| Cg-Gal4 ; E(z)^731^  >UAS-abd-A | 1 | 114 | | 108 | 94.74 | 93.86 | 2.79 |
|  | 2 | 103 | | 99 | 96.12 |  |  |
|  | 3 | 108 | | 98 | 90.74 |  |  |
| Cg-Gal4 ; brm^2^ >UAS-abd-A | 1 | 125 | | 124 | 99.20 | 97.96 | 1.97 |
|  | 2 | 116 | | 111 | 95.69 |  |  |
|  | 3 | 98 | | 97 | 98.98 |  |  |
| Cg-Gal4 ; Trl^R85^ >UAS-abd-A | 1 | 104 | | 81 | 77.88 | 76.97 | 8.84 |
|  | 2 | 96 | | 65 | 67.71 |  |  |
|  | 3 | 109 | | 93 | 85.32 |  |  |

**Supplementary Table 9:** Effect of PcG and trxG proteins mutations on tumor phenotype

**Supplementary Table 10:** Pupal lethality in abd-A over-expression in background of PcG and trxG mutants.

| **Genotype** | **Experiment** | **Total pupae** | **Dead pupae** | **Lethality (%)** | **Avg.** | **Std. Dev.** |
| --- | --- | --- | --- | --- | --- | --- |
| Cg-Gal4/+ | 1 | 50 | 0 | 0.00 | 0 | 0.00 |
|  | 2 | 50 | 0 | 0.00 |  |  |
|  | 3 | 50 | 0 | 0.00 |  |  |
| Cg-Gal4>UAS-abd-A | 1 | 43 | 42 | 97.67 | 99 | 1.34 |
|  | 2 | 48 | 48 | 100.00 |  |  |
|  | 3 | 25 | 25 | 100.00 |  |  |
| Su(z)2^1.a1^,Cg-Gal4>UAS-abd-A | 1 | 24 | 23 | 95.83 | 95 | 1.57 |
|  | 2 | 31 | 29 | 93.55 |  |  |
|  | 3 | 29 | 28 | 96.55 |  |  |
| Psc^1^, Cg-Gal4>UAS-abd-A | 1 | 36 | 16 | 44.44 | 47 | 2.79 |
|  | 2 | 32 | 15 | 46.88 |  |  |
|  | 3 | 38 | 19 | 50.00 |  |  |
| esc^2^, Cg-Gal4>UAS-abd-A | 1 | 17 | 13 | 76.47 | 66 | 8.65 |
|  | 2 | 18 | 11 | 61.11 |  |  |
|  | 3 | 21 | 13 | 61.90 |  |  |
| Cg-Gal4 ; Pc^1^>UAS-abd-A | 1 | 42 | 42 | 100.00 | 100 | 0.00 |
|  | 2 | 23 | 23 | 100.00 |  |  |
|  | 3 | 21 | 21 | 100.00 |  |  |
| Cg-Gal4 ;Su(z)12^3^e^1^red^1^ >UAS-abd-A | 1 | 40 | 40 | 100.00 | 100 | 0.00 |
|  | 2 | 43 | 43 | 100.00 |  |  |
|  | 3 | 45 | 45 | 100.00 |  |  |
| Cg-Gal4; E(z)^731^ >UAS-abd-A | 1 | 32 | 31 | 96.88 | 98 | 1.62 |
|  | 2 | 44 | 43 | 97.73 |  |  |
|  | 3 | 23 | 23 | 100.00 |  |  |
| Cg-Gal4 ; brm^2^>UAS-abd-A | 1 | 24 | 21 | 87.50 | 89 | 1.28 |
|  | 2 | 17 | 15 | 88.24 |  |  |
|  | 3 | 30 | 27 | 90.00 |  |  |
| Cg-Gal4; Trl^R85^>UAS-abd-A | 1 | 34 | 28 | 82.35 | 79 | 3.30 |
|  | 2 | 29 | 23 | 79.31 |  |  |
|  | 3 | 33 | 25 | 75.76 |  |  |

**Supplementary table 11A) Relative hemocyte specific GFP levels when *Hml-Gal4, He gal4* and *cg-Gal4* are used to drive the expression of *UAS-mcd8-GFP***

| Hml>gfp | Mean | SD | He>gfp | Mean | SD | cg>gfp | Mean | SD |
| --- | --- | --- | --- | --- | --- | --- | --- | --- |
| 2576 | 2630.4 | 370.5513 | 4546 | 5189.5 | 1477.769 | 4418 | 4437.8 | 687.7895 |
| 2771 |  |  | 5019 |  |  | 3334 |  |  |
| 2460 |  |  | 2699 |  |  | 5306 |  |  |
| 2800 |  |  | 8403 |  |  | 5255 |  |  |
| 1896 |  |  | 5325 |  |  | 4128 |  |  |
| 2462 |  |  | 4465 |  |  | 4603 |  |  |
| 2893 |  |  | 4297 |  |  | 3193 |  |  |
| 2885 |  |  | 5841 |  |  | 5006 |  |  |
| 3303 |  |  | 6807 |  |  | 4742 |  |  |
| 2258 |  |  | 4493 |  |  | 4393 |  |  |

**Supplementary table 11B) Relative fat-body expression of GFP when driven by *cg-Gal4* and *Lsp2-Gal4***

| cg>GFP fat body | Mean | SD | Lsp2>GFP fat body | Mean | SD |
| --- | --- | --- | --- | --- | --- |
| 16189 | 15296.9 | 1104.751 | 15383 | 14087.7 | 1940.076 |
| 15271 |  |  | 14753 |  |  |
| 15913 |  |  | 16101 |  |  |
| 14913 |  |  | 13094 |  |  |
| 15395 |  |  | 16216 |  |  |
| 15543 |  |  | 15913 |  |  |
| 15991 |  |  | 13094 |  |  |
| 12279 |  |  | 9752 |  |  |
| 15092 |  |  | 14164 |  |  |
| 16383 |  |  | 12407 |  |  |

**Supplementary table 12: Number of PH3+ hemocytes**

| **cg-Gal4>UAS-abd-A** | **+** | **Pc1** | **Psc1** | **E(Z)731** | **esc2** | **Su(z)123** | **TrlR85** | **brm2** |
| --- | --- | --- | --- | --- | --- | --- | --- | --- |
|  | 5 | 2 | 3 | 12 | 5 | 10 | 6 | 6 |
|  | 7 | 8 | 5 | 10 | 8 | 13 | 4 | 3 |
|  | 5 | 4 | 6 | 3 | 8 | 4 | 10 | 7 |
|  | 4 | 9 | 9 | 6 | 4 | 2 | 13 | 5 |
|  | 5 | 12 | 8 | 8 | 6 | 7 | 4 | 4 |
|  | 4 | 7 | 1 | 7 | 4 | 8 | 1 | 4 |
|  | 10 | 9 | 4 | 4 | 6 | 12 | 12 | 8 |
|  | 13 | 10 | 2 | 6 | 2 | 6 | 5 | 5 |
|  | 9 | 6 | 8 | 3 | 7 | 14 | 8 | 10 |
|  | 8 | 14 | 10 | 2 | 4 | 5 | 7 | 4 |
|  | 7 | 13 | 7 | 9 | 12 | 1 | 5 | 3 |
|  | 8 | 9 | 12 | 11 | 6 | 6 | 8 | 7 |
| Average | 7.08333 | 8.58333 | 6.25 | 6.75 | 6 | 7.3333 | 6.9166 | 5.5 |
| SD | 2.5967 | 3.3778 | 3.21778 | 3.19178 | 2.48327 | 4.02768 | 3.35306 | 2.06155 |
