# Supplementary material for: Drosophila Hox genes induce melanized pseudo-tumors when misexpressed in hemocytes
Source: Sci Rep. 2021 Jan 19;11:1838. doi: 10.1038/s41598-021-81472-5 (PMC7815749; doi:10.1038/s41598-021-81472-5)
Supplement: Supplementary file 4 — Supplementary Information 4. [file 41598_2021_81472_MOESM4_ESM.pdf]

***Drosophila* Hox genes induce melanized pseudo-tumors when misexpressed in hemocytes**

Titus Ponrathnam, Ravina Saini, Sofia Banu and Rakesh K Mishra*

Centre for Cellular and Molecular Biology, Hyderabad, 500007, Telangana, India

Supplementary Figures

Supplementary Figure 1A

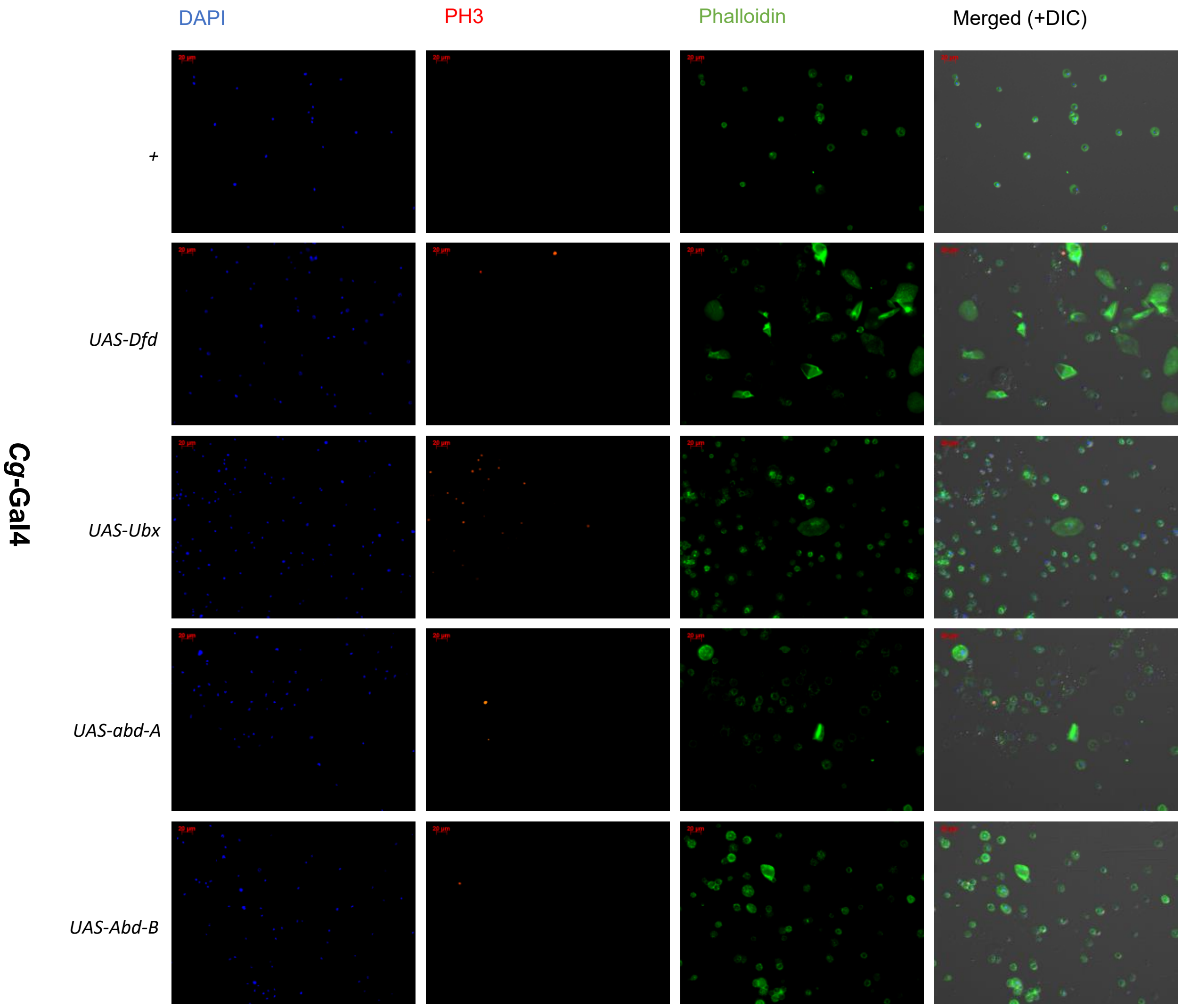

Supplementary Figure 1: PH3 Staining of circulating blood cells. Hox genes induce cell autonomous proliferation when expressed under the drivers (A) *cg*-Gal4, (B) *He*-Gal4, (C) *Hml*-Gal4, but not when expressed in the fatbody under *Lsp2*-Gal4 (D)

Supplementary Figure 1B

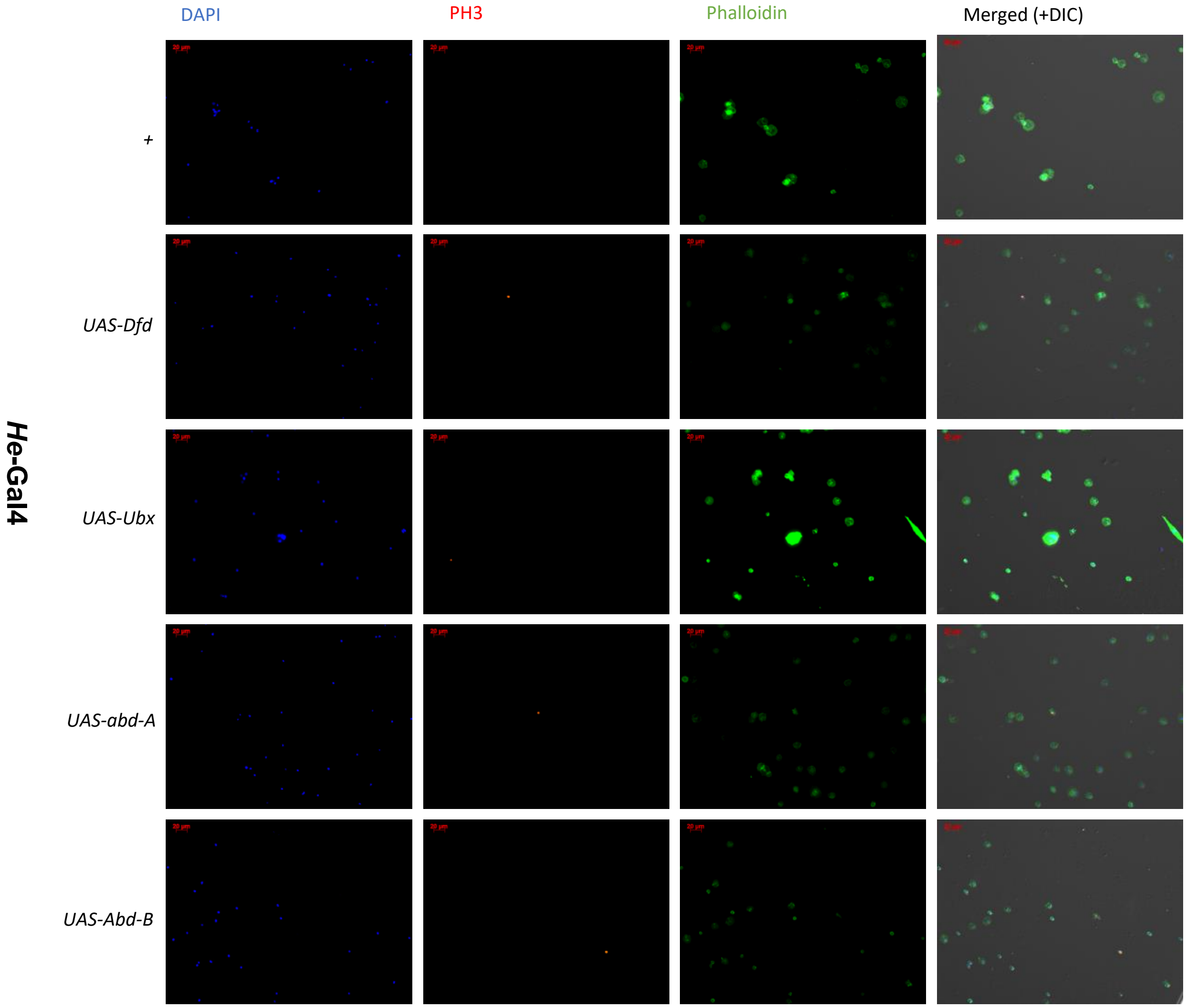

Supplementary Figure 1C

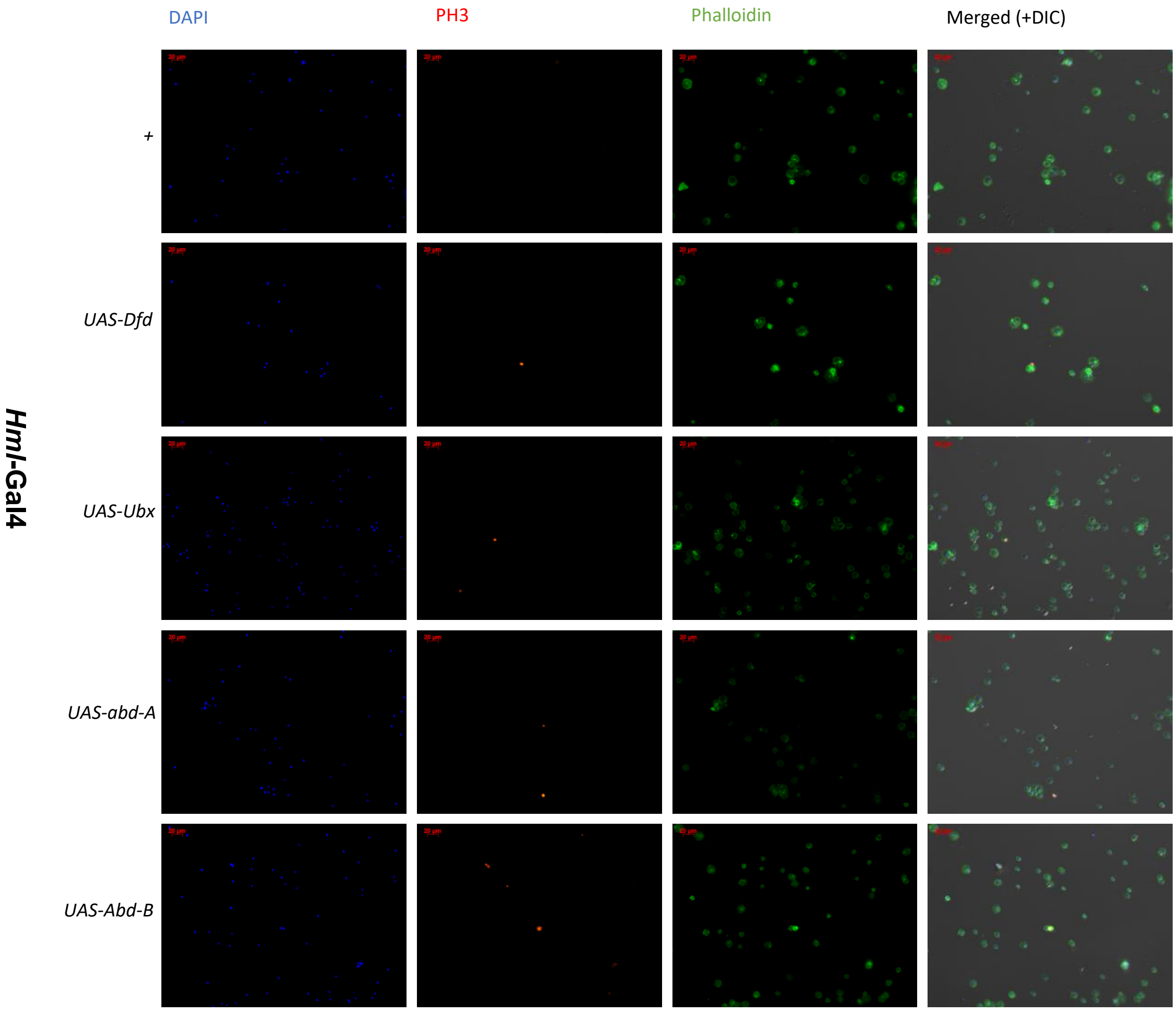

Supplementary Figure 1D

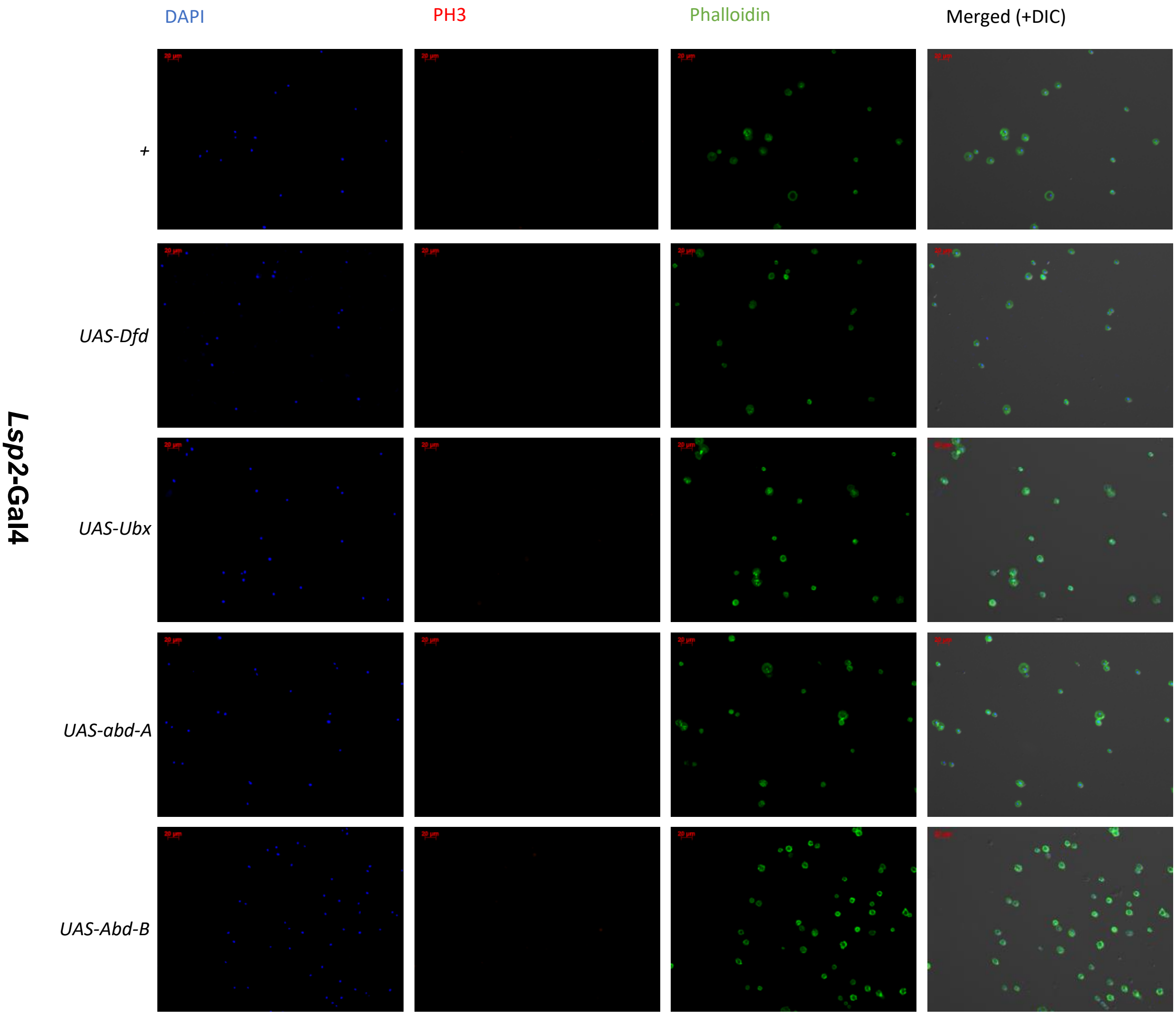

Supplementary figure :2A

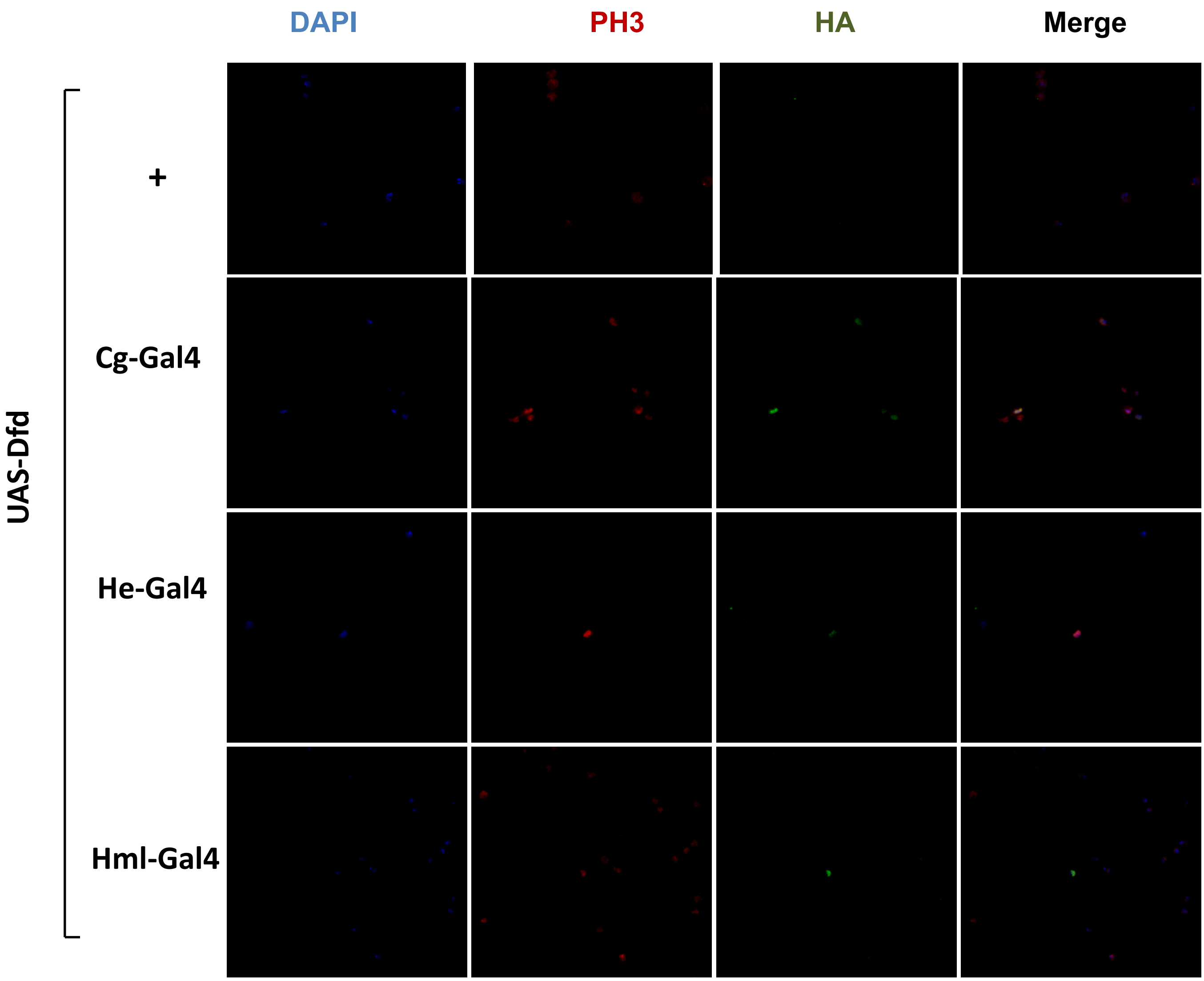

Supplementary figure 2 : Anti-HA staining for Hox over-expression and PH3 co-localization of circulating blood cell. Different Hox genes (A) UAS-DFd, (B) UAS-Ubx (C) UAS-abdA and (D) UAS-AbdB are stained with anti-HA (green) . PH3 (red) and DAPI (blue).

Supplementary figure :2B

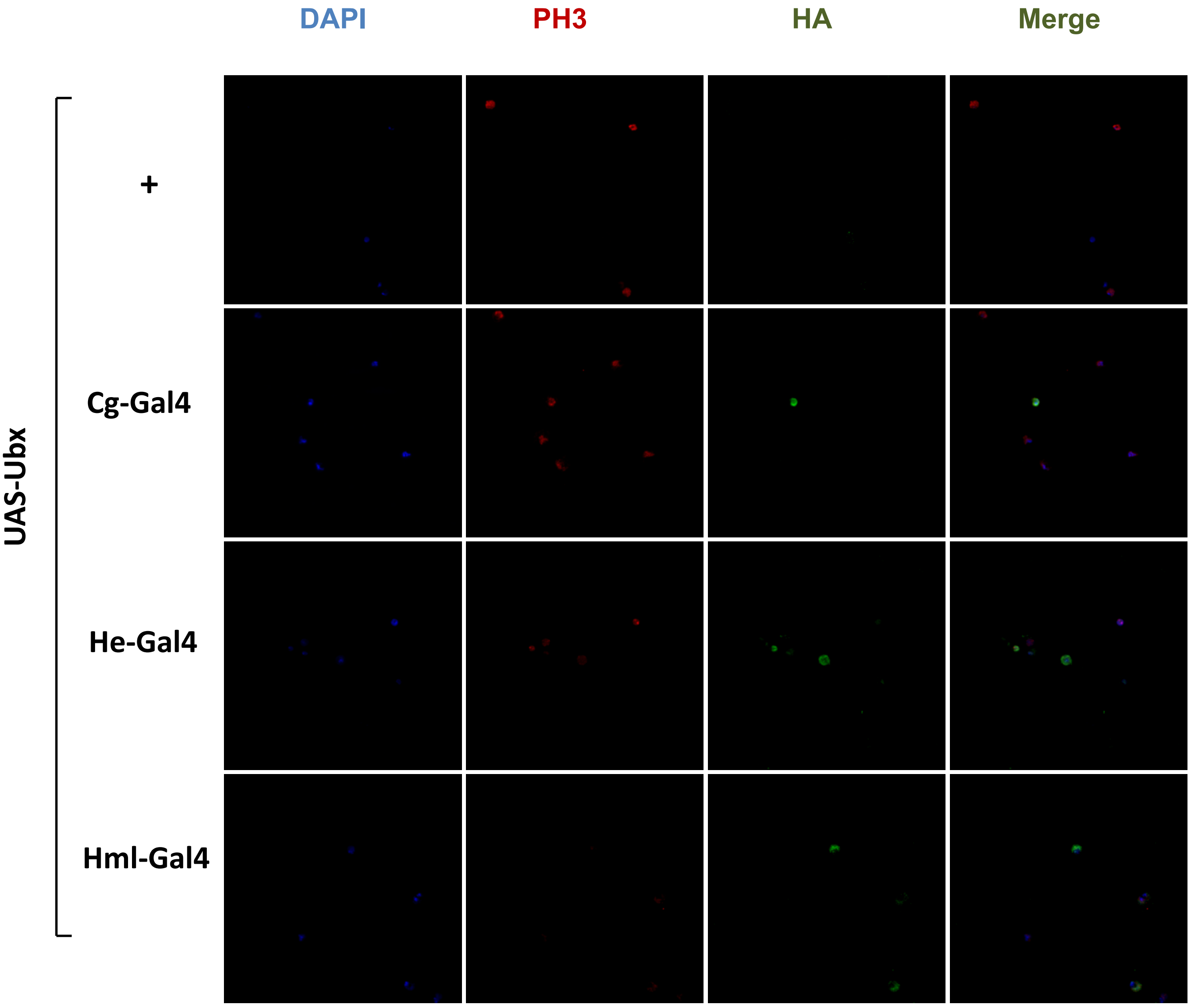

Supplementary figure :2C

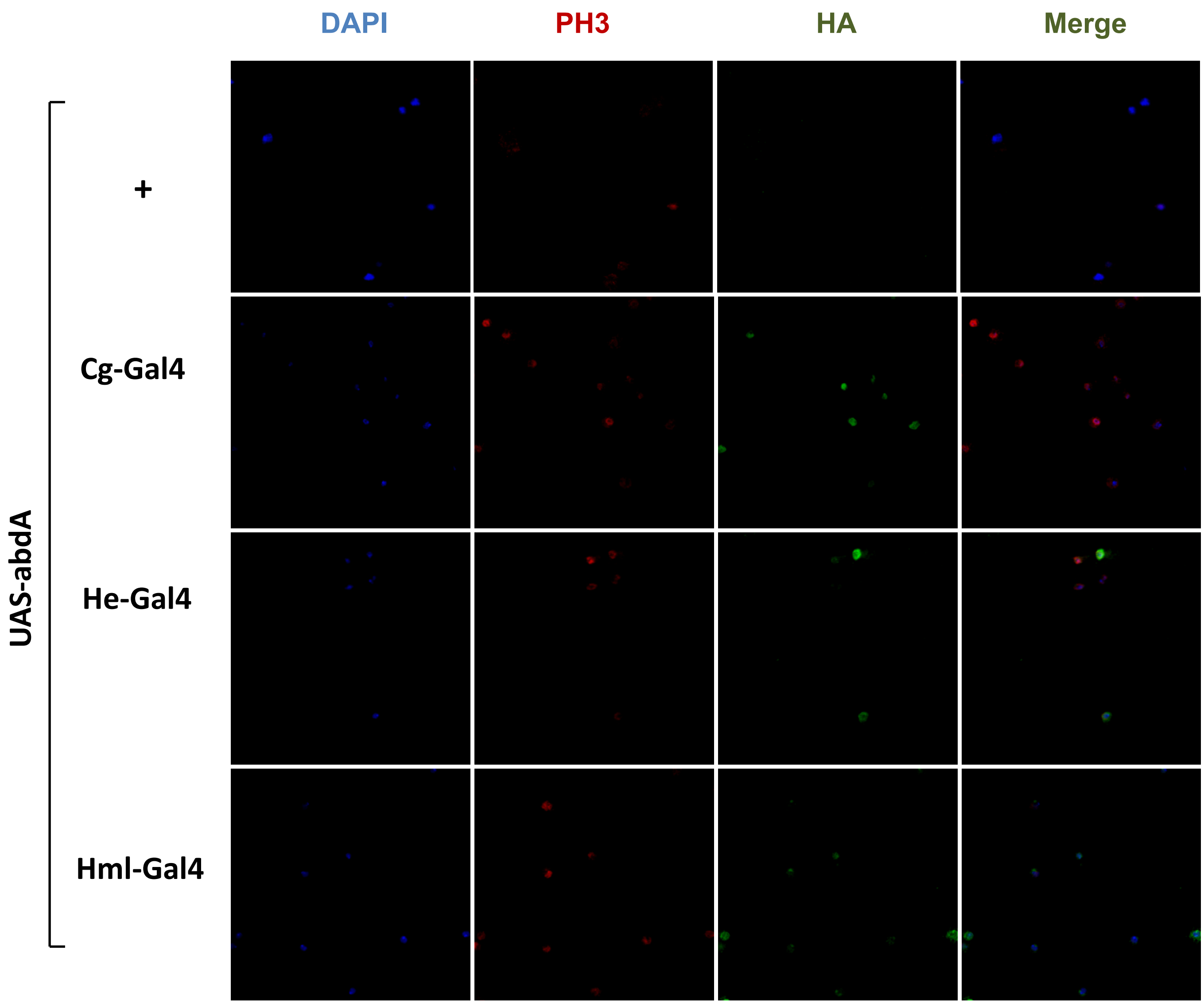

Supplementary figure :2D

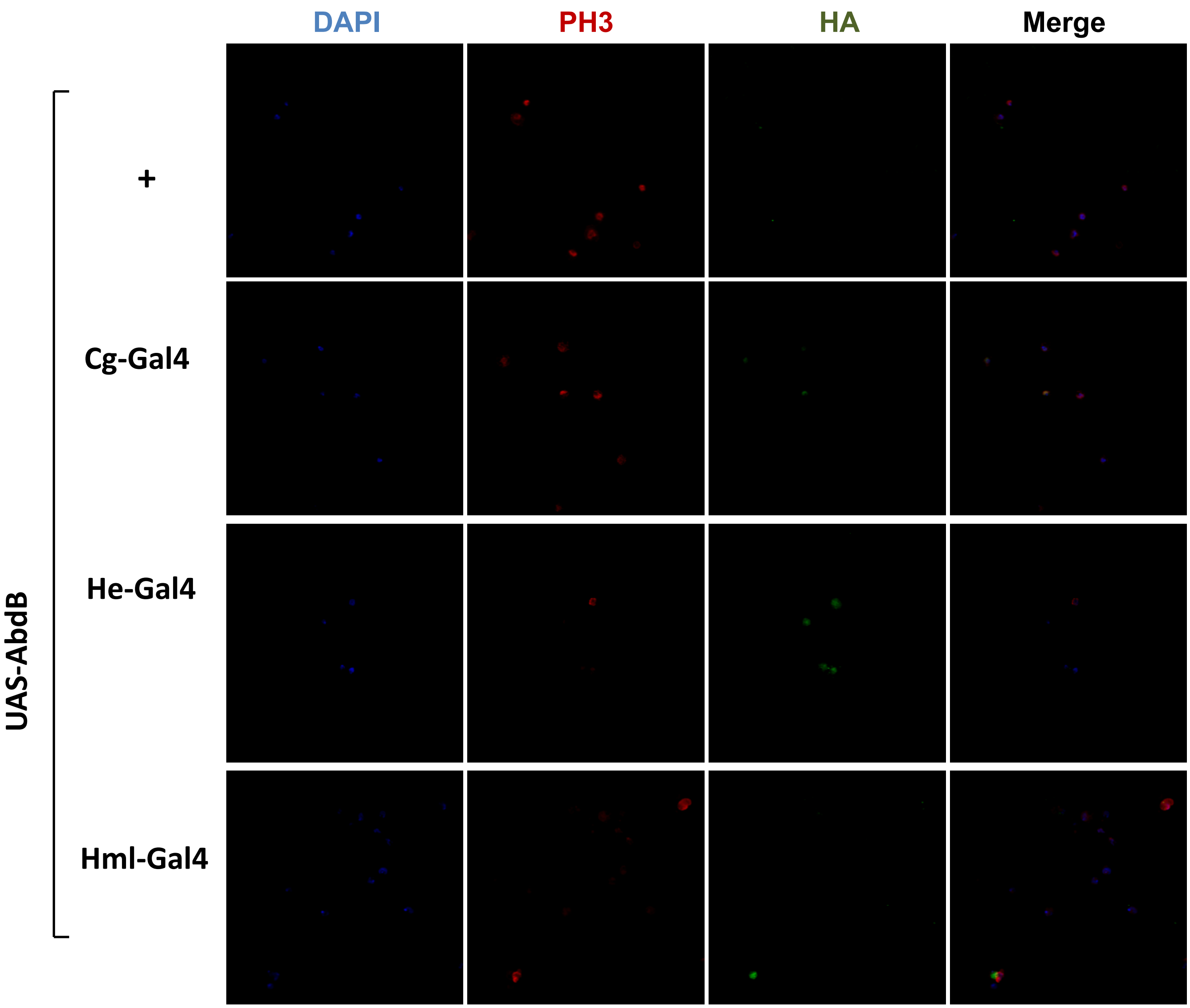

Supplementary figure :3A

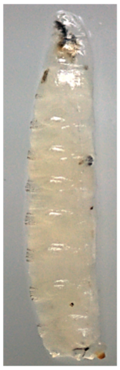

Larva with Small melanized body

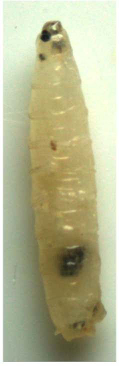

Larva with large melanized body

Supplementary figure :3B

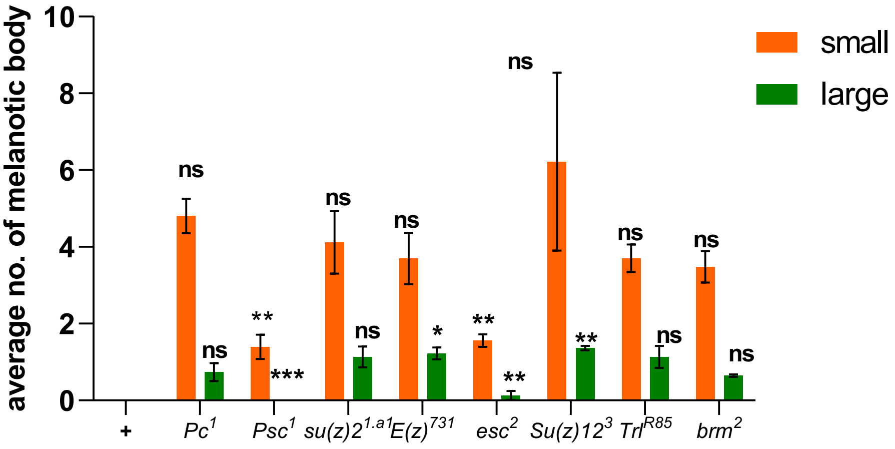

**Cg-Gal4**	+	+	+	+	+	+	+	+	+
**UAS-abdA**	+	+	+	+	+	+	+	+	+

Supplementary figure 3 : Severity of the melanized psudo-tumor phenotype in PcG and trxG mutant background. Small and large tumors were counted in phenotype showing individuals. (A) larvae with small and large tumors.

Supplementary figure :4A

Blood - abdA overexpression vs Control

**Regulation**	**Genes**
Upregulated	444
Downregulated	2290

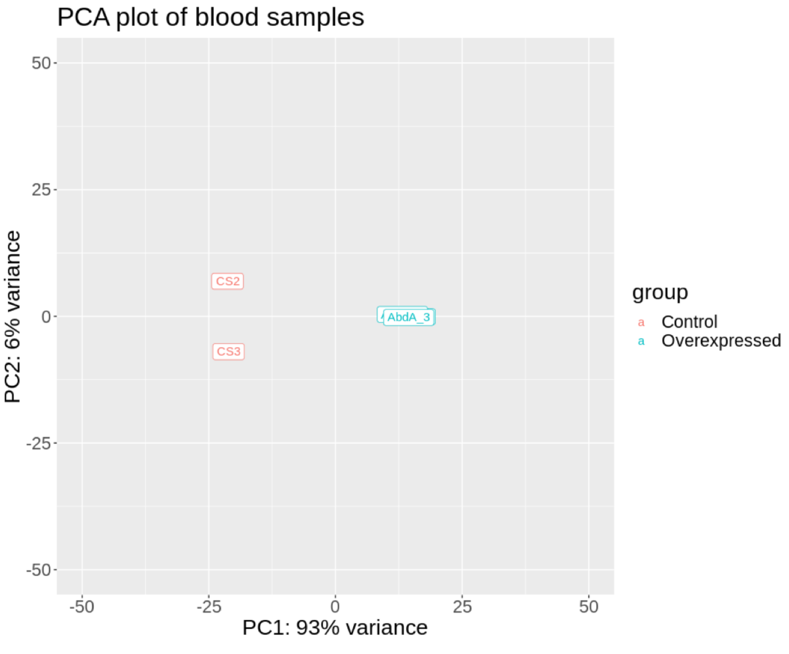

Supplementary Figure 4. a) PCA plot of the RNA seq replicates **b)** Gene ontology distribution of upregulated genes **c)** Gene Ontology distribution of downregulated genes.

Supplementary figure :4B

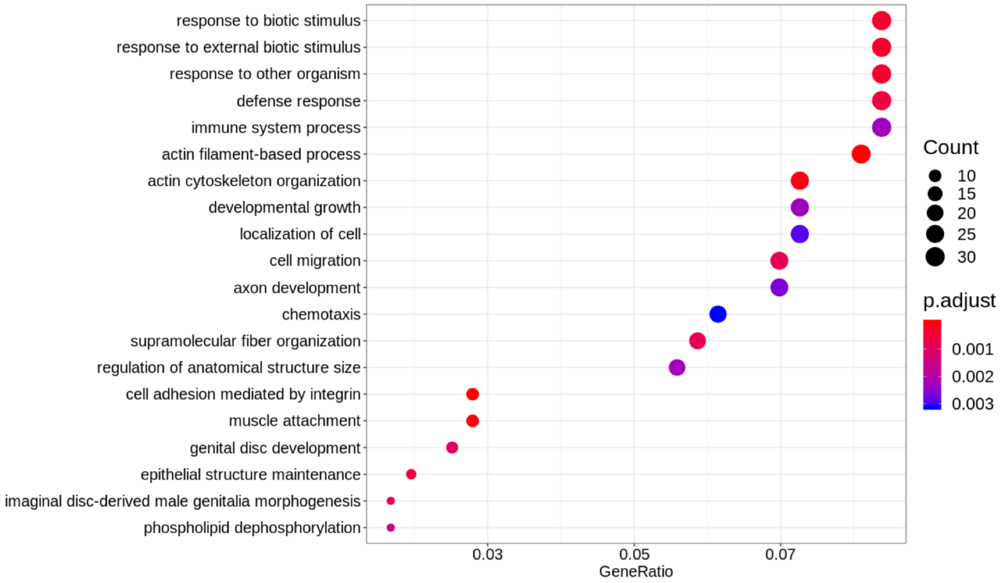

Gene Ontology enrichment - Upregulated genes - Blood

Supplementary figure :4C

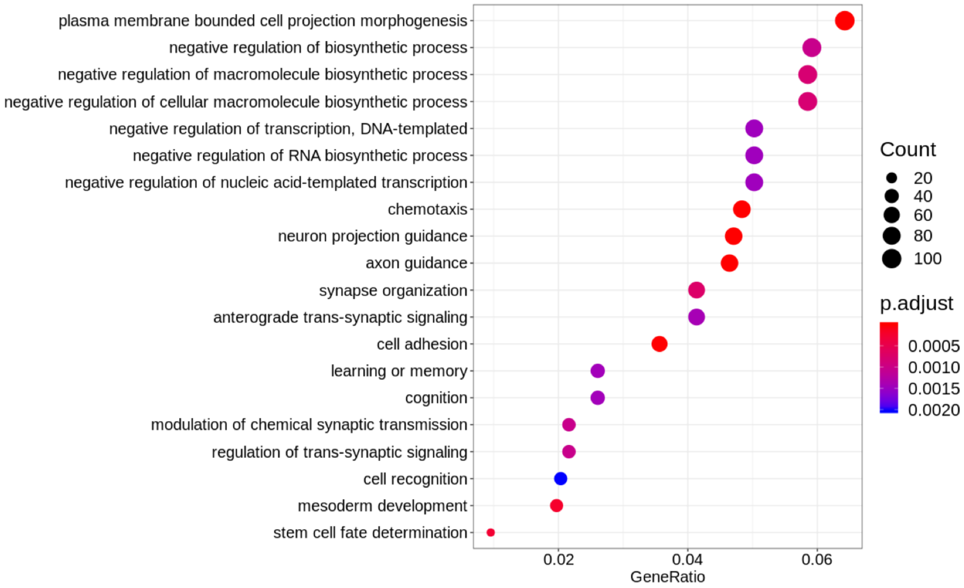

Gene Ontology enrichment - Downregulated genes - Blood
